# Rare earth elements from waste

**DOI:** 10.1126/sciadv.abm3132

**Published:** 2022-02-09

**Authors:** Bing Deng, Xin Wang, Duy Xuan Luong, Robert A. Carter, Zhe Wang, Mason B. Tomson, James M. Tour

**Affiliations:** 1 Department of Chemistry, Rice University, Houston, TX 77005, USA.; 2 Department of Civil and Environmental Engineering, Rice University, Houston, TX 77005, USA.; 3 Department of Materials Science and NanoEngineering, Rice University, Houston, TX 77005, USA.; 4 Smalley-Curl Institute, NanoCarbon Center and the Welch Institute for Advanced Materials, Rice University, Houston, TX 77005, USA.

## Abstract

Rare earth elements (REEs) are critical materials in electronics and clean technologies. With the diminishing of easily accessible minerals for mining, the REE recovery from waste is an alternative toward a circular economy. Present methods for REE recovery suffer from lengthy purifications, low extractability, and high wastewater streams. Here, we report an ultrafast electrothermal process (~3000°C, ~1 s) based on flash Joule heating (FJH) for activating wastes to improve REE extractability. FJH thermally degrades or reduces the hard-to-dissolve REE species to components with high thermodynamic solubility, leading to ~2× increase in leachability and high recovery yields using diluted acid (e.g., 0.1 M HCl). The activation strategy is feasible for various wastes including coal fly ash, bauxite residue, and electronic waste. The rapid FJH process is energy-efficient with a low electrical energy consumption of 600 kWh ton^−1^. The potential for this route to be rapidly scaled is outlined.

## Teaser

The ultrafast flash Joule heating process greatly increases the recovery yield of rare earth elements from industrial wastes.

## INTRODUCTION

Rare earth elements (REEs) are strategic resources in modern electronics, clean energy, and automotive industries (*1*). Concentrated aqueous acid leaching of the REE minerals, followed by biphasic solvent extraction, has been the dominant scheme for REE mass production (*1*). However, the resource- and pollution-intensive production has a large environmental footprint, where the degrative environmental cost reached $14.8 billion in 2015, warranting a search for a sustainable solution (*2*). As the easily accessible REE minerals diminish, the extraction of REE from industrial wastes has gained much attention (*3*). The applicable secondary wastes include coal fly ash (CFA) (*4*–*9*); bauxite residue (BR; which was formerly called red mud) (*10*–*12*), which results from bauxite processing for aluminum production; and electronic waste (e-waste) (*13*–*15*) from consumer electronics and electric vehicles. The reuse of these wastes, in turn, reduces the environmental burden of their disposal (*8*). However, the total REE contents in these secondary wastes are usually less than those in REE minerals, and the recycling yields are still extremely low, which exacerbate the quest to establish a circular economic program (*4*).

Taking CFA as an example, it is the by-product of coal combustion with an annual production rate of ~750 million tons worldwide (*8*). CFA has an average total REE content of ~500 parts per million (ppm), which is variable based on the geological origin of the feed coals (*4*, *9*). The acid-extractable REE content, however, is usually much smaller and highly dependent on the CFA feeds. For example, Taggart *et al.* (*4*) reported the HNO_3_ extractability of REE ranging from 1.6 to 93.2% with a median value of ~30% from major U.S. power plants, or 7.4 to 372 ppm with a median value of ~127 ppm (*4*). REE extractability in CFA depends on the REE species, such as oxides, phosphates (churchite, xenotime, monazite, etc.), apatite, zircon, and glass phases (*7*). The low REE extractabilities in most CFA resources are attributed to the large ratios of hard-to-dissolve REE species such as REE phosphates, zircon, and glass phases (*7*).

Optimizing acid leaching processes could, to some extent, improve the extractability by using highly concentrated mineral acids, such as 15 M HNO_3_ at 85° to 90°C for an extractability of 70% (*4*) and 12 M HCl at 85°C for an extractability of 35 to 100%, depending on the feeds (*16*). The use of concentrated acid, however, inevitably increases the cost of extraction and the disposal burden. Chemical or thermal pretreatment of the CFA before acid leaching contributes to achieving high REE recovery (*17*, *18*). For example, a total REE recovery of 88% is achieved by the NaOH hydrothermal treatment followed by acid leaching (*17*). Alkali roasting using NaOH leads to a recovery yield of >90% (*18*). However, those pretreatment processes are usually lengthy and energy-intensive, which greatly reduce the profit margin and incentive. Hence, a rapid and energy-efficient pretreatment is imperative for the REE recovery from waste products.

Recently, electrical heating has been emerging as an ultrafast, high-temperature, and energy-efficient heating manner for materials synthesis and processing. For example, the high-temperature shock technique uses rapid pulsed current for the ultrafast synthesis of functional nanomaterials (*19*–*22*). The ultrahigh-temperature sintering based on continuous current input is proposed for ceramic sintering and screening in seconds (*23*). Our group developed the flash Joule heating (FJH) process for conversion of carbon-containing sources into flash graphene (*24*). In addition to the materials synthesis capability (*25*), the FJH process has been demonstrated as an efficient technique for sustainable management of carbon-rich wastes (*26*), including the conversion of plastic waste (*27*) and rubber waste (*28*) to graphene.

Here, we reported the ultrafast electrothermal process based on FJH to activate the secondary wastes to improve the acid extractability of REE simply using a mild acid such as 0.1 M HCl. A pulsed voltage in 1 second brings the raw materials to a temperature of ~3000°C, leading to the thermal decomposition of the hard-to-dissolve REE phosphates in CFA into highly soluble REE oxides, and the carbothermic reduction of REE components to highly reactive REE metals. The activation process enables the increase in REE recovery yields to ~206% for class F-type CFA (CFA-F) and ~187% for class C-type CFA (CFA-C) compared to directly leaching the raw materials with more concentrated acids. The activation strategy is feasible for various secondary wastes, as demonstrated by CFA, BR, and e-waste. The rapid FJH process is scalable and highly energy-efficient with a low electrical energy consumption of 600 kilowatt-hour (kWh) ton^−1^ or $12 ton^−1^, enabling a profit percentage of >10×. Among all REE, five of them (Y, Nd, Eu, Tb, and Dy) are most critical based on their need for clean energy devices and their supply risk (*29*). The percentages of extractable critical REE in the FJH-activated CFA, BR, and e-waste are two to three times higher than those in some of the most concentrated ores in the world, thus underscoring the usefulness of these local sources that are abundant, requiring no additional mining, and categorized as toxic waste and problematic to stockpile.

## RESULTS

### Acid-extractable REE content in CFA

**Fig. 1.**
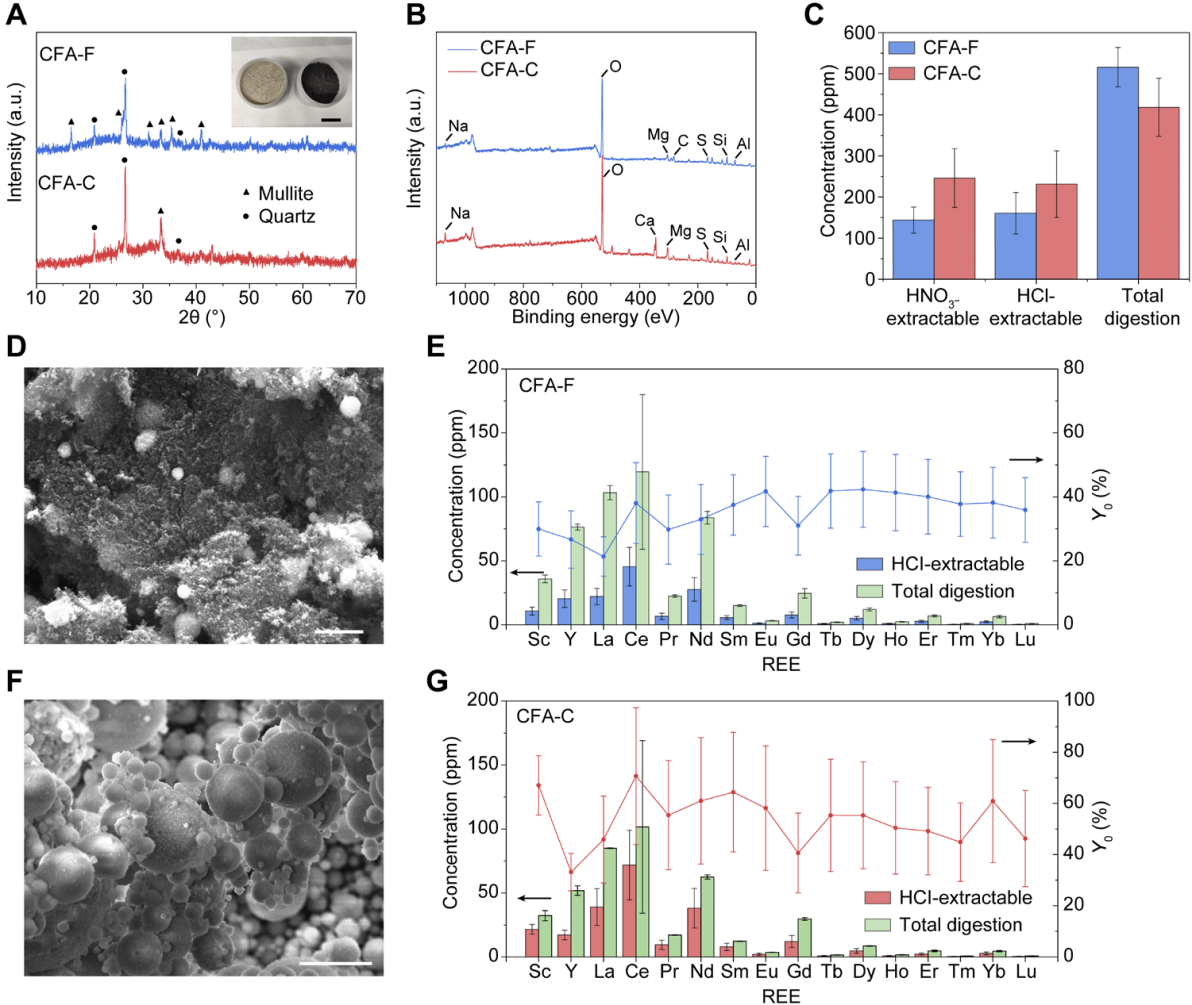
Acid-extractable REE content in CFA. (**A**) XRD patterns of CFA-F and CFA-C. Inset: Picture of CFA-C (left) and CFA-F (right). Scale bar, 4 cm. a.u., arbitrary units. (**B**) XPS full spectra of CFA-F and CFA-C. (**C**) Concentration of total REE in CFA-F and CFA-C by HNO_3_ leaching (15 M, 85°C), HCl leaching (1 M, 85°C), and total quantification. (**D**) SEM image of CFA-F. Scale bar, 2 μm. (**E**) HCl-extractable REE contents (1 M, 85°C) and total quantification of REE in CFA-F and the recovery yield (*Y*_0_) of REE. (**F**) SEM image of CFA-C. Scale bar, 5 μm. (**G**) HCl-extractable REE contents (1 M, 85°C) and total quantification of REE in CFA-C and the recovery yield (*Y*_0_) of REE. All error bars in (C), (E), and (G) represent the SD, where *N* = 3.

There are two types of CFA categorized by the chemical composition, CFA-F, with the total content of SiO_2_, Al_2_O_3_, and Fe_2_O_3_ >70 weight % (wt %), and CFA-C, with a higher abundance of CaO (*7*). In this work, CFA-F is collected from the Appalachian Basin (App), and CFA-C is collected from the Powder River Basin (PRB) (*4*), both in the United States. CFA is composed of primary amorphous phases (60 to 90%) (*6*), and the remaining crystalline materials include mainly quartz and mullite, as shown by the x-ray diffraction (XRD) patterns (Fig. 1A). In addition to the enrichment of Ca in CFA-C, elemental analyses by x-ray photoelectron spectroscopy (XPS) (Fig. 1B) and energy-dispersive x-ray spectroscopy (fig. S1) show a high C content in CFA-F, which might be caused by the incomplete combustion of coal feeds. The high C content in CFA-F is also evident by the large weight loss at ~700°C by thermal gravimetric analysis (TGA) (fig. S2).

The total quantification of REE in CFA was done by the hydrofluoric acid (HF):HNO_3_ digestion method (see details in Materials and Methods) (*4*). The total REE content, *c*_total_(CFA Raw), is 516 ± 48 mg kg^−1^ for CFA-F and 418 ± 71 mg kg^−1^ for CFA-C (Fig. 1C). The CFA from App has a higher REE content than that from PRB, consistent with a previous report (*4*). Acid-leachable REE contents from CFA raw materials, *c*_0_(CFA Raw), were measured by using 1 M HCl or 15 M HNO_3_ (see details in Materials and Methods) (*4*, *9*). For CFA-F, the HNO_3_- and HCl-extractable REE contents are 144 ± 32 mg kg^−1^ and 160 ± 50 mg kg^−1^ (Fig. 1C), respectively, corresponding to the REE extractability (*Y*_0_) of ~28 and ~31%, respectively. For CFA-C, the HNO_3_- and HCl-extractable REE contents are 246 ± 71 mg kg^−1^ and 231 ± 81 mg kg^−1^ (Fig. 1C), respectively, corresponding to the REE extractability of ~59 and ~55%, respectively. It is concluded that the acid concentration has limited effect on the REE leachability once it is >1 M. Hence, in later experiments, we used the 1 M HCl leaching as the standard protocol.

The acid extractability of REE from CFA-C is higher than that from CFA-F. This is consistent with a previous report (*7*), which attributes the higher extractability to the higher content of easy-to-dissolve REE species like REE oxides in CFA-C. The morphology image by scanning electron microscopy (SEM) of CFA-F is shown in Fig. 1D, and the high carbon content could retard the accessibility of aqueous acids to REE-bearing species, leading to the low extractability ranging from 21 to 42% for individual REE (Fig. 1E). In contrast, CFA-C is composed of fine, uncovered spheric particles (Fig. 1F), which benefits the acid leaching process, leading to a relatively higher extractability ranging from 33 to 67% for individual REE (Fig. 1G).

### Improved recovery yield of REE from CFA by electrothermal activation

**Fig. 2.**
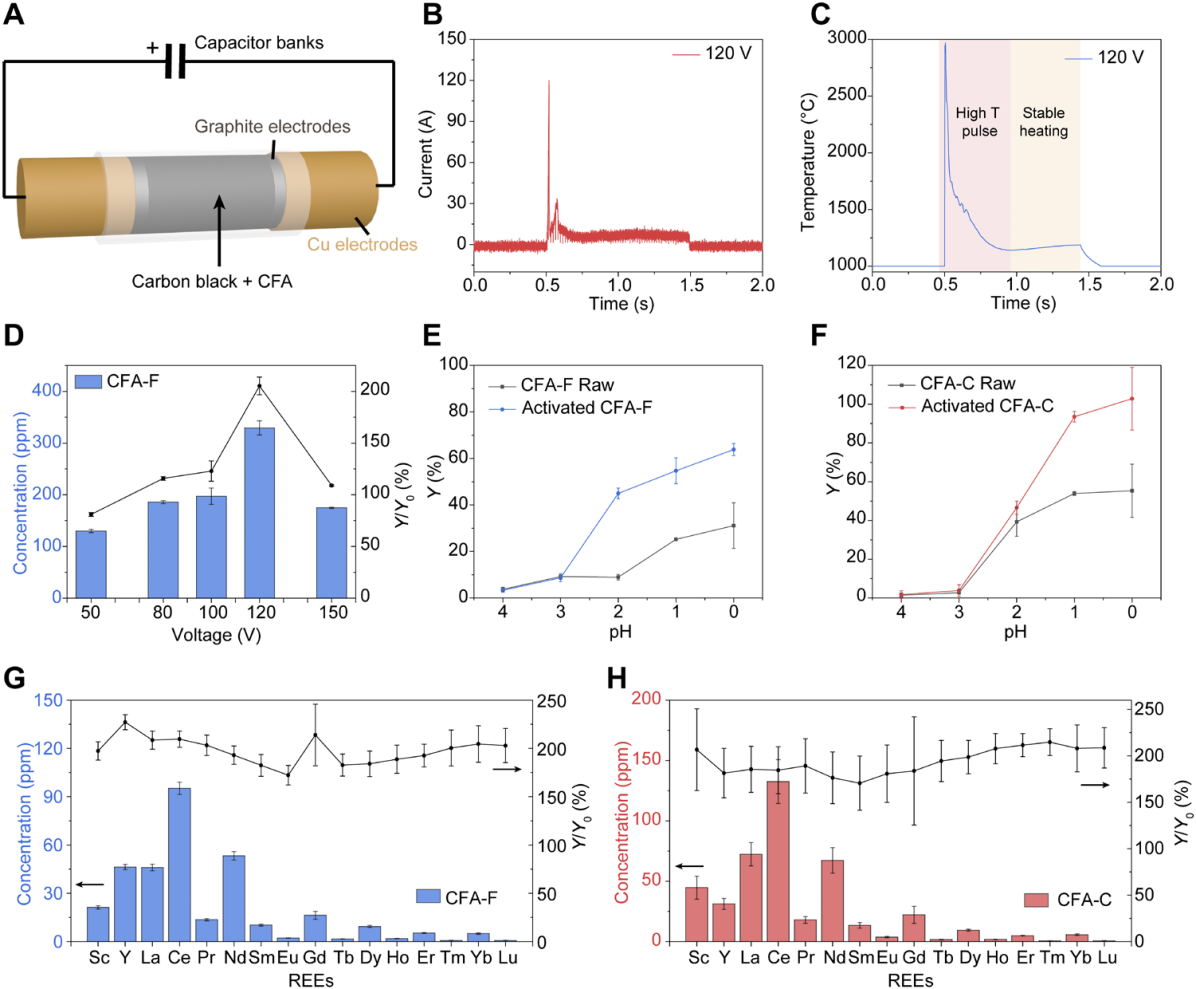
Improved recovery yield of REE from CFA by electrothermal activation. (**A**) Scheme of the FJH of CFA. (**B**) Current curve with the FJH condition of 120 V and 1 s. (**C**) Real-time temperature measurement with the FJH condition of 120 V and 1 s. (**D**) Relationship between HCl-leachable REE contents (1 M, 85°C) from activated CFA-F, increase in recovery yield (*Y*/*Y*_0_), and the FJH voltages. (**E**) pH-dependent REE leachability from the CFA-F raw materials and activated CFA-F. (**F**) pH-dependent leachability of REE from the CFA-C raw materials and activated CFA-C. (**G**) HCl-leachable individual REE contents (1 M, 85°C) from activated CFA-C and the increase in recovery yield. (**H**) HCl-leachable individual REE contents (1 M, 85°C) from activated CFA-F and the increase in recovery yield. *Y*_0_ represents the REE recovery yield by HCl leaching the CFA raw materials, and *Y* represents the REE recovery yield by HCl leaching the activated CFA. All error bars in (D) to (H) represent the SD, where *N* = 3.

In our electrothermal activation process by FJH, CFA raw materials were first mixed with carbon black (CB), which serves as the conductive additive. The mixture of CFA and CB (~30 wt % CB) was loaded inside a quartz tube between two graphite electrodes (Fig. 2A and fig. S3). The resistance (*R*) of the sample was tunable by adjusting the compressive force between the two electrodes that were connected to a capacitance bank of 60 mF (fig. S3). The sample was brought to a high temperature by high-voltage discharging of the capacitors. The detailed experimental parameters are shown in table S1. In a typical discharging process with FJH voltage of 120 V, *R* of 1 ohm, and discharging time of 1 s, the current curve passing through the sample was recorded with the peak current at ~120 A, followed by a current plateau at ~7 A (Fig. 2B). The corresponding real-time temperature curve exhibits a peak temperature up to ~3000°C, followed by the stable heating at ~1150°C (Fig. 2C). The temperature map of the sample during the FJH shows that the temperature distribution is uniform throughout the entire sample without an obvious gradient (fig. S4), demonstrating that the FJH has a homogeneous heating capability. Because the sample has a much larger resistance than the graphite electrodes, the heat generated by discharging is mainly imposed onto the sample (fig. S5). Thus, the FJH setup has a good durability even though such a high temperature can be achieved. The obtained solid after the FJH is termed as activated CFA (fig. S6). The acid-leachable REE content from the activated CFA, *c*(activated CFA), was measured by the 1 M HCl leaching procedure. The recovery yield of REE from the activated CFA (*Y*) was calculated and compared with that of the CFA raw materials (*Y*_0_) (text S1).

A series of FJH voltage ranging from 50 to 150 V were applied (Fig. 2D). At ~120 V, the HCl-leachable content of total REE (1 M HCl, 85°C) from the activated CFA-F is improved to 329 ± 14 mg kg^−1^ (Fig. 2D). This corresponds to the recovery yield of *Y* ~ 64%, representing an increase to ~206% over that of the CFA-F raw materials (*Y*_0_ ~ 31%). The pH-dependent leaching dynamics of REE from CFA-F raw materials and activated CFA-F were investigated (Fig. 2E). In general, the yield is reduced as the acid pH increases. The recovery yield of REE from the activated CFA-F remains *Y* ~ 45% at pH 2 (or 0.01 M HCl), substantially higher than that of the CFA raw materials at the same leaching condition (*Y*_0_ ~ 9% at pH 2), and even under a much higher acid concentration (*Y*_0_ ~ 31% at pH 0). For CFA-C, under the optimized FJH condition, the acid leachability of REE from the activated CFA-C is measured to be *Y* ~ 103% using the HCl leaching procedure (1 M HCl, 85°C) (Fig. 2F), corresponding to ~187% of that from the CFA-C raw materials (*Y*_0_ ~ 55%). Even using a dilute acid (pH 1 or 0.1 M HCl), the recovery yield of REE from the activated CFA-C remains *Y* ~ 94%, substantially higher than that of the CFA-C raw materials (*Y*_0_ ~ 54%). This would render far more manageable wastewater streams.

For individual REE, with the FJH activation process, the acid leachability is improved ranging from ~170 to ~230% for CFA-F (Fig. 2G) and from ~170 to ~210% for CFA-C (Fig. 2H) using the same leaching procedure (1 M HCl, 85°C). Similar improvements were realized using a dilute acid leaching (0.1 M HCl, 85°C) (fig. S7). Some deviations among the different REE are observed, which could be attributed to the inhomogeneous distribution of REE in CFA (Fig. 1, E and F), as well as the different activation degree among REE (as will be discussed in detail later). As control, the REE content in CB was measured using the same digestion method (fig. S8). The total REE content in CB is ~5 mg kg^−1^, corresponding to ~1% of that in CFA. Hence, the use of CB will not induce notable error into our measurement. In practical applications, the CB could be substituted with anthracite coal or any other inexpensive sources of mildly conductive carbon, but the REE content in that source needs to be considered in yield calculations.

### Mechanism of the improved REE extractability

We then investigated the mechanism of the improved REE leachability by the electrothermal activation process. The REE speciation and distribution in CFA determine the REE extractability. REE phosphate, including monazite and xenotime, is one of the primary counterions of REE in coal (*7*, *30*). REE phosphates are rather stable components, and no melting or thermal dissociation of them occurs up to ~2000°C in air (*31*, *32*). The coal-fire combustion temperature typically ranges from 1300° to 1700°C (*30*). As a result, the REE-bearing trace phases, including monazite and xenotime, persist in CFA (*33*, *34*). The REE could also be partitioned and encapsulated into the glass fraction of CFA by diffusion into the melt (e.g., aluminosilicates) formed at the coal boiler temperature (*35*). Those hard-to-dissolve REE phosphates and glass phases are detrimental for REE extraction (*7*), while REE oxides and carbonates in CFA are relatively easier to extract by acid leaching.

**Fig. 3.**
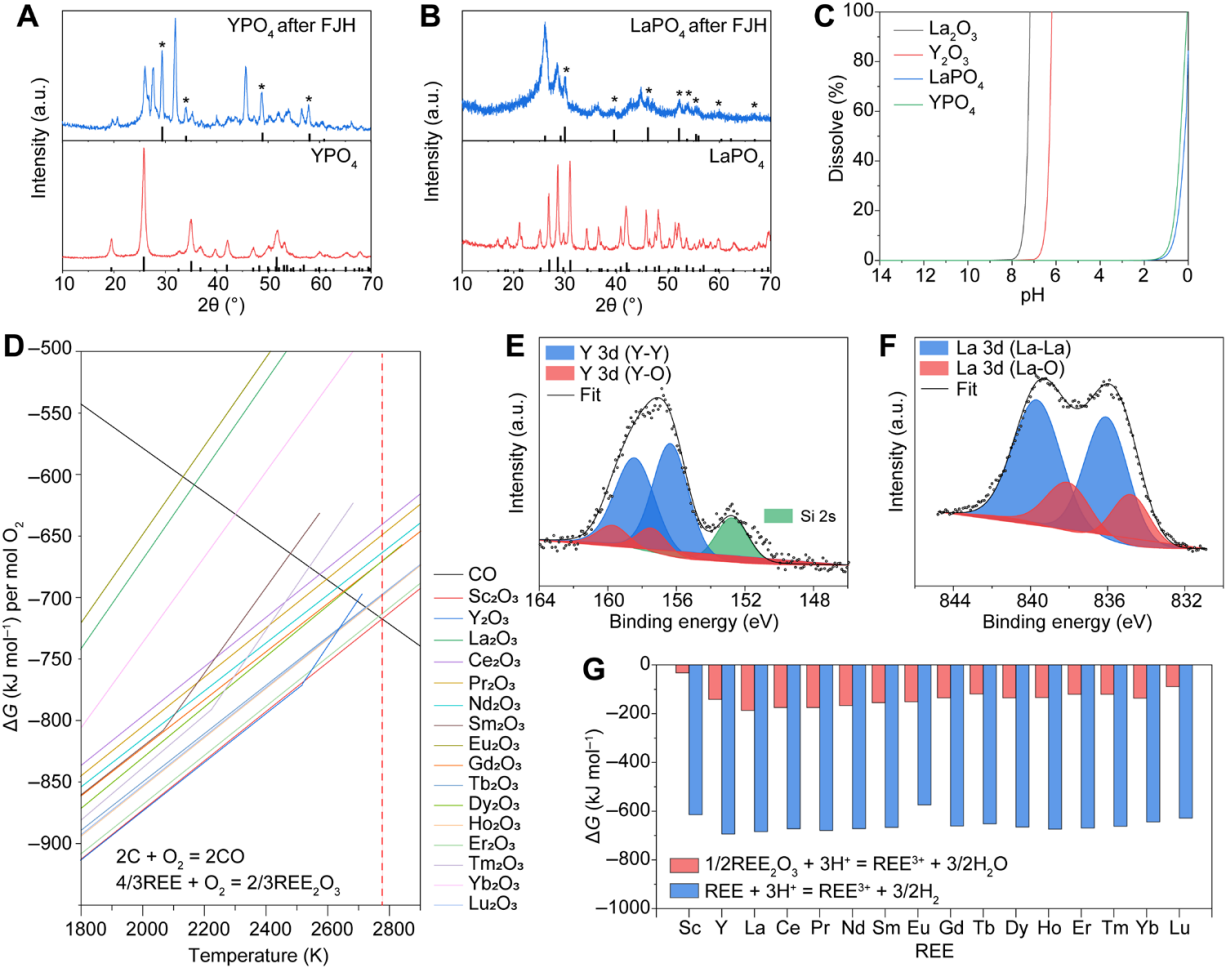
Mechanism of the improved REE extractability by the electrothermal activation. (**A**) XRD patterns of YPO_4_ (bottom) with reference PDF (YPO_4_, #11-0254) and YPO_4_ after FJH (top) with reference PDF (Y_2_O_3_, #43-0661). (**B**) XRD patterns of LaPO_4_ (bottom) with reference PDF (LaPO_4_, #35-0731) and LaPO_4_ after FJH (top) with reference PDF (La_2_O_3_, #05-0602). The asterisks denote the diffraction peaks from La_2_O_3_. (**C**) Calculated dissolution curves of Y_2_O_3_, YPO_4_, La_2_O_3_, and LaPO_4_ with a mass of 1 g in 100-ml solution. Cl^−^ is used to balance the charge. (**D**) Ellingham diagram of carbon monoxide and REE oxides. The red dashed line denotes the temperature to reduce Sc_2_O_3_. (**E**) XPS fine spectrum of Y_2_O_3_ after FJH. The Si signal might be from the quartz tube during FJH. (**F**) XPS fine spectrum of La_2_O_3_ after FJH. (**G**) Gibbs free energy change of the REE oxide and REE metal dissolution reactions in acid.

The high temperature of ~3000°C generated by the FJH process, which is substantially higher than the coal boiler temperature, could thermally degrade the REE species. The thermal decomposition temperatures of REE phosphates are calculated to be between ~2600° and ~2900°C under standard conditions (table S2). Experimentally, yttrium phosphate (YPO_4_) and lanthanum phosphate (LaPO_4_) were used as representatives for REE phosphates. As shown in Fig. 3A, after FJH of the YPO_4_ precursor, the Y_2_O_3_ phase is identified in the product. Similarly, LaPO_4_ is thermally decomposed to Y_2_O_3_ after the FJH process (Fig. 3B). While we not seeking to identify all new material phases accessed during FJH, the REE oxides have much higher solubility (log_10_*K*_sp_ of 5 to 33) than REE phosphates (log_10_*K*_sp_ of −27 to −24) (table S3). To further provide insight into the solubility of REE phosphates and oxides, we calculated the dissolution curves as a function of pH (Fig. 3C). It is found that LaPO_4_ and YPO_4_ show notable solubility only when pH approaches 0, while the oxide counterparts readily dissolve at a low acidity with pH ~6. This partially explains the observed pH-dependent REE leaching dynamics that higher REE leachabilities are achieved for the activated CFA than the raw materials using dilute acid (Fig. 2, E and F).

In addition to the thermal decomposition of REE phosphates, the ultrahigh temperature achieved by FJH could also trigger the thermal reduction of REE compounds. According to the calculated Ellingham diagram (Fig. 3D), the carbothermic reduction temperatures of REE oxides are estimated to be between ~1900°C (for Eu_2_O_3_) and ~2500°C (for Sc_2_O_3_). The FJH at ~120 V generates a temperature up to ~3000°C (Fig. 2C), which permits the reduction of all REE oxides. Y_2_O_3_ and La_2_O_3_ were used as representatives to verify the carbothermic reduction of REE oxides by the FJH process. The fitting of the XPS fine spectrum of Y_2_O_3_ after FJH shows four peaks (Fig. 3E and table S4). The peaks at 157.5 and 159.6 eV are assigned to 3d_5/2_ and 3d_3/2_ of Y in Y_2_O_3_, respectively (fig. S9A) (*36*), and the peaks at 156.4 and 158.5 eV are assigned to 3d_5/2_ and 3d_3/2_ of Y in Y(0), respectively (*37*). The XPS analysis proved the reduction of Y_2_O_3_ to Y metal by the FJH process, while the small ratio of Y_2_O_3_ might be from the surface oxidation. Similarly, the fitting of XPS fine spectra of La_2_O_3_ precursor (fig. S9B) and La_2_O_3_ after FJH (Fig. 3F and table S4) verifies the reduction of La_2_O_3_ to La metal (*38*, *39*). The reduced REE species with low oxidation state are highly active materials that readily react with even pure water (*40*). The calculated Gibbs free energy change (Δ*G*) values for the REE metal dissolution reaction are much more negative than those of REE oxides (Fig. 3G and table S3), demonstrating a much larger thermodynamic solubility of REE metals than for their oxide counterparts.

The above analysis suggests that the required temperature for the thermal activation is >2600°C for thermal decomposition of REE phosphates and >2500°C for carbothermic reduction of REE oxides, which also provides insight into the voltage-dependent REE leachability (Fig. 2D). An FJH voltage of ≥120 V is essential for achieving a temperature of >2000°C (fig. S10), while a voltage of <100 V has limited effect on the REE leachability. Nevertheless, too high of an FJH voltage, ≥150 V, leads to a prolonged high temperature of >3000°C, which could, in turn, result in the evaporative loss of the REE during the FJH process. The observed different activation degree for different REE by the FJH process (Fig. 2, G and H) could be attributed to their different intrinsic thermodynamic properties, specifically the varied thermal decomposition temperature of REE phosphates (table S2) and carbothermic reduction temperature of REE oxides (Fig. 3D).

In addition to speciation, the REE distribution also affects the extractability, where the REEs encapsulated in or distributed throughout the glass phases are hard to dissolve (*7*). The FJH permits an ultrafast heating and rapid cooling (>10^4^ K s^−1^; Fig. 2C), which would induce thermal stress and cracking of the glass phases in CFA, contributing to the improved leachability.

### Generality of the electrothermal activation process

**Fig. 4.**
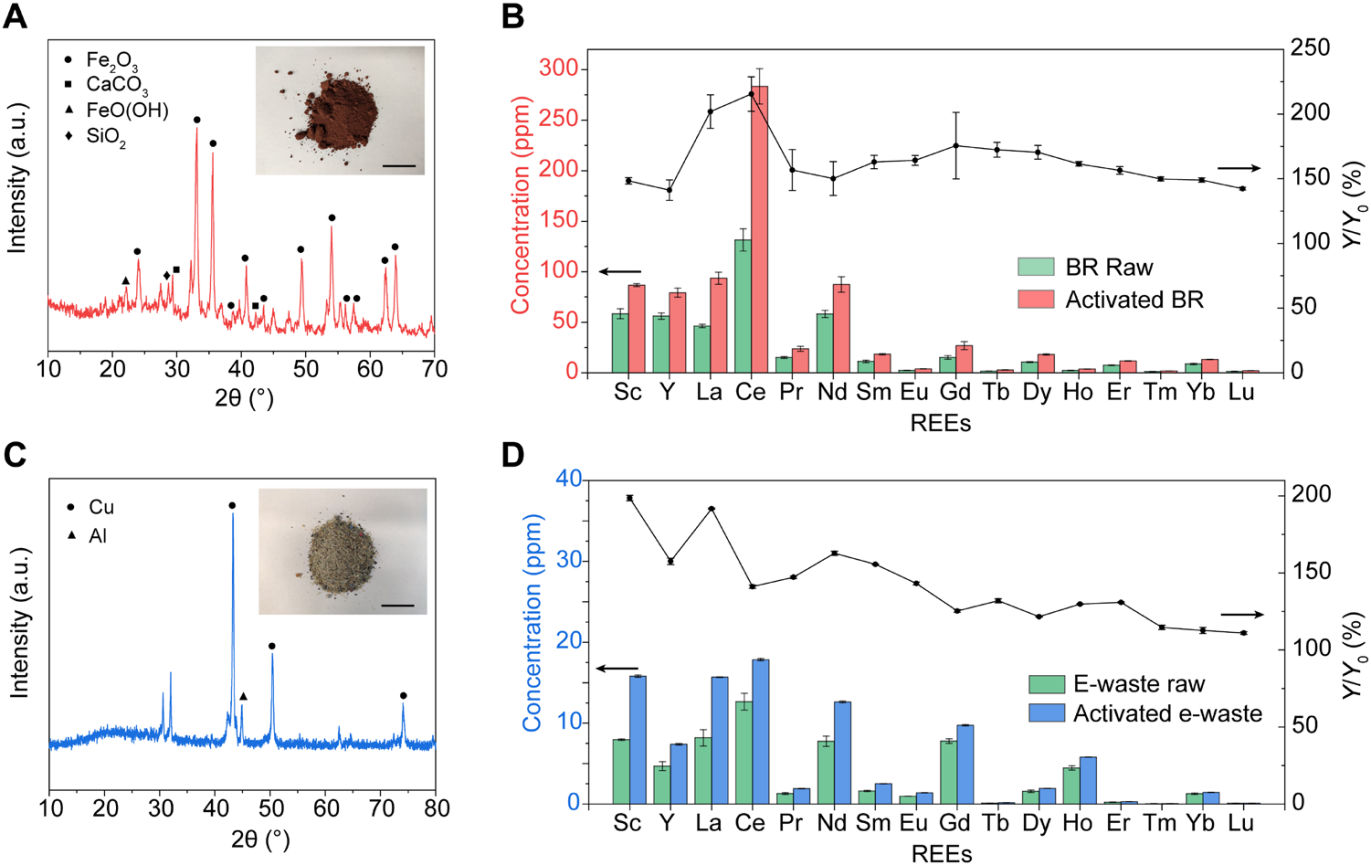
Recovery of REE from BR and e-waste. (**A**) XRD pattern of BR. Inset: Picture of BR. Scale bar, 5 cm. (**B**) Acid-leachable individual REE contents (0.5 M HNO_3_) from BR raw materials and the 120-V FJH-activated BR and the increase in recovery yield (*Y*/*Y*_0_). (**C**) XRD pattern of e-waste. Inset: Picture of e-waste ground to powders. Scale bar, 5 cm. (**D**) Acid leachable REE contents (1 M HCl) from e-waste raw materials and the 50-V FJH-activated e-waste and the increase in recovery yield. In (B) and (D), *Y*_0_ represents the REE recovery yield by acid leaching the raw materials, and *Y* represents the REE recovery yield by acid leaching the activated materials. All error bars in (B) and (D) represent the SD, where *N* = 3.

The electrothermal activation process could be extended to other waste products for REE recovery, including BR (*10*–*12*) and e-waste (*13*–*15*). BR is the waste product of the Bayer process for alumina production. BR is one of the most abundant industrial wastes, with 3 billion tons already stored in waste ponds and an additional 150 million tons produced each year, yet just ~3% is currently recycled (*41*). BR contains a notable amount of REE, for example, a total REE content of ~1000 ppm is found in BR from MYTILINEOS “Aluminum of Greece” (*10*). The BR is a dried powder with fine particle size (fig. S11) and has major components including Fe_2_O_3_, CaCO_3_, FeO(OH), and SiO_2_ (Fig. 4A). The REE in BR was extracted by a direct leaching process using 0.5 M HNO_3_ (see details in Materials and Methods) (*42*). The HNO_3_-extractable REE content from BR raw materials is 428 ± 9 mg kg^−1^ (fig. S12 and Fig. 4B). Similar to CFA, the REE extractability of the BR after the electrothermal activation process is also dependent on the FJH voltage (fig. S12A). At the optimized FJH voltage of 120 V, the HNO_3_-extractable REE content increased to 757 ± 30 mg kg^−1^ (fig. S12B), corresponding to *Y*/*Y*_0_ ~ 177% of that from the BR raw materials (Fig. 4B). The mechanism of the improvement of REE extractability from BR by the FJH process is presumed to be similar to that of CFA (Fig. 3), because phosphate is also one of the dominant counterions for BR (*43*).

We also applied the FJH strategy for activating e-waste. More than 40 million tons of e-waste are produced globally each year due to the rapid upgrade of personal electronics, with <20% being recycled (*44*). REEs are widely used in electronics in permanent magnets (*14*), batteries (*13*), and capacitors (*45*). In turn, the recovery of REE from high-grade e-waste has its economic feasibility compared to REE mining from ores. The e-waste used in this work is a printed circuit board (PCB) from a discarded computer. As shown in Fig. 4C, the abundant metals in e-waste include Cu and Al, which are mainly used as the interconnects. The REE in the PCB waste was extracted by 1 M HCl leaching process at 85°C (see details in Materials and Methods). The acid-leachable REE content is 61 ± 4 mg kg^−1^ from the e-waste raw materials (fig. S13). After the activation process at an optimized voltage of 50 V (fig. S13), the HCl-extractable REE content is increased to 94.6 ± 0.2 mg kg^−1^, corresponding to *Y*/*Y*_0_ ~ 156% of that from the e-waste raw materials (Fig. 4D and fig. S13). Different from CFA or BR, the REE species in e-waste are usually in the form of easy-to-dissolve REE metals or oxides (*45*). However, the REEs are usually embedded into the matrix materials because of the laminated configuration of the electronics, which could hinder the REE extraction by the hydrometallurgical process (fig. S14). The FJH process could expose the REE species by cracking the matrices, accelerating the leaching rate and extent of metal extraction (fig. S14).

## DISCUSSION

Among all the REEs, five of them (Y, Nd, Eu, Tb, and Dy) are considered most critical based on the importance to clean energy and supply risk (*4*, *29*). The percentages of HCl-extractable critical REE in activated CFA-F and CFA-C are ~34 and ~26%, respectively (fig. S15, A and B). The percentages of the critical REE extracted from the CFA raw materials and the activated CFA are almost identical (fig. S15, A and B), indicating that the FJH process shows no discriminative activation performance to different REE. In addition, the percentages of extractable critical REE in activated BR and e-waste are ~26 and ~29%, respectively (fig. S15, C and D). The critical REE percentages in these wastes are considerably higher than those in conventional ores (typically <15%) (*4*). For example, the world’s largest REE deposit at Bayan Obo in China has a critical REE percentage of <10% (*46*). The higher percentage of critical REE in wastes compared to conventional minerals represents another major advantage of the recycling scheme.

The FJH process for REE recovery is scalable (text S2 and figs. S16 and S17). According to the theoretical analysis, to maintain a constant temperature when scaling up the sample mass per batch, we could increase the FJH voltage or the total capacitance of the capacitor bank (text S2). In our research laboratory, a production rate of >10 kg day^−1^ by the batch-by-batch process has already been realized (fig. S16). The FJH process could presumably be integrated into the continuous production manner for further automation (fig. S17). The major challenge for further scaling up the FJH process for REE recovery would be the design and construction of larger-scale equipment. This might be addressed by the application of the well-established high-voltage or even ultrahigh-voltage technologies in industry (*47*, *48*). In addition, the alternating current (AC) FJH (AC-FJH) could be introduced to complement the present direct current (DC) supply (*27*). The ongoing commercial scaling of the FJH process to tons per day paves the way for future REE recovery from large-scale waste products (text S2).

We investigated the economics because the profit margin is often the sustainer of recycling. Because of the direct sample heating feature, short duration, and rapid heating/cooling rate, the FJH process is highly energy-efficient with a low electrical energy consumption of 600 kWh ton^−1^ or $12 ton^−1^, enabling a profit percentage of >10× compared to directly leaching the raw materials (text S3). For further refining, the removal of dissolved impurities, including mainly Al, Si, Fe, Ca, and Mg, in the REE-containing leachate and subsequent separation are needed (text S4). We observed that the content ratio of REE and impurity [*c*(REE)/*c*(Impurity)] in the leachate is improved with the FJH process in most cases (figs. S18 to S20), indicating that the FJH process would also be beneficial for the subsequent REE separation.

Because monazite, (Ce, La, Y, Th)PO_4_, and xenotime, YPO_4_, are the main commercial sources for REE production (*1*), the proposed activation strategy could also work for the REE mining to improve the leachability from REE ores. Commercially, alkaline digestion (70% NaOH, 140° to 150°C) is the main leaching technology for monazite (*49*) or acid baking (concentrated H_2_SO_4_, 200°C) for monazite and xenotime (*50*). The FJH strategy could be faster and less dependent on the use of concentrated bases and acids. Existing individual elemental separation technologies, such as solvent extraction and ion exchange (*51*), can be exploited to work with the REE mixtures obtained by FJH because these are often less contaminated than those generated through traditional mining methods (text S4).

## MATERIALS AND METHODS

### Materials

The chemicals used are La(NO_3_)_3_·6H_2_O (≥99 wt %, Fluka Analytical), La_2_O_3_ (99.9%, MilliporeSigma), Y_2_O_3_ (99.99%, MilliporeSigma), YPO_4_ (99.99%, Alfa Aesar), CB (Cabot, BP-2000), H_3_PO_4_ (85 wt %, Sigma-Aldrich), HCl [37 wt %, ≥99.999% trace metal grade for inductively coupled plasma (ICP) analysis, MilliporeSigma], HNO_3_ (67 to 70 wt %, trace metal grade for ICP analysis, Fisher Chemical), H_2_SO_4_ [98 wt %, GR ACS grade (guaranteed reagent meeting American Chemical Society standards), MilliporeSigma], and HF (48 wt %, ≥99.99%, trace metal grade for ICP analysis, MilliporeSigma). LaPO_4_ was synthesized by direct precipitation (*52*). Concentrated H_3_PO_4_ was heated to a temperature of 150°C, and La(NO_3_)_3_·6H_2_O was added slowly to allow the emission of NO*_x_* gas. Milky white solid particles precipitated within 30 min, which were then diluted and collected by filtering using a sand core funnel (class F). The white solids were dried in an oven (100°C) for 2 hours. The CFA-F samples were collected from App, and CFA-C samples were collected from PRB and provided to our laboratory [see Acknowledgments (*4*)]. The BR was collected from MYTILINEOS S.A. in Greece and provided to our laboratory (see Acknowledgments). The PCB waste was from a discarded computer. The PCB was cut into small pieces and then ground into powders using a hammer grinder (Dade, DF-15).

### FJH system and process

The electrical diagram of the FJH system is shown in fig. S3A. The picture of the FJH system is shown in fig. S3B. The description of each electrical component of the system is listed in the Supplementary Materials. In a typical experiment, the secondary wastes (CFA, BR, or e-waste) were mixed with CB with a mass ratio of 2:1 by using a ball miller (MSE Supplies, PWV1-0.4L). The CB served as the conductive additive. The 200-mg mixture (133 mg of waste and 67 mg of CB) was added into a quartz tube (inner diameter of 8 mm and outer diameter of 12 mm). The resistance was controlled by compressing the two electrodes. The samples were loaded into the jig (fig. S3C), and the electrodes were connected to the capacitor bank. The capacitor bank with a total capacitance of 60 mF was charged by a DC supply. A relay with programmable milliseconds-level relay was used to control the discharging time. The detailed experimental parameters are summarized in table S1. After the FJH, the samples were rapidly cooled to room temperature. For the scaling up of the process to the gram scale, a larger FJH system with a total capacitance of 0.624 F was built and used (fig. S3D). A larger quartz tube (inner diameter of 16 mm and outer diameter of 20 mm) and a larger reaction jig were used (fig. S3E). Safety caution: There is a risk of electrical shock without proper operation. Safety glass should be worn to protect the eyes from the bright light emission during the FJH process. The “one hand rule” should be obeyed, and thick rubber gloves extending to the elbows should be used. More safety implementations were shown in fig. S3.

### Sample digestion, leaching, and ICP-MS/ICP-OES measurement

For the CFA samples, acid-extractable REE content measurement, including HNO_3_ and HCl, and total REE quantification were conducted (*4*). For HNO_3_ leaching, ~50-mg samples (CFA raw materials) were digested in 10-ml concentrated HNO_3_ (15 M) at 85°C for 4 hours. The sample was filtered using a sand core funnel (class F) and diluted using ultrapure water for ICP mass spectrometry (MS) measurement. For HCl leaching, ~50-mg samples (CFA raw materials or the activated CFA by FJH) were digested in ~10-ml HCl (1 M) at 85°C for 4 hours. After digestion, the sample was filtered using a sand core funnel (class F) and diluted to ~20 ml using ultrapure water for ICP-MS measurement. The pH-dependent leaching dynamics were investigated by using 0.1, 0.01, 0.001, and 0.0001 M HCl at pH 1 to 4, respectively, as the leaching agents. Samples (~50 mg; CFA raw materials or the activated CFA by FJH) were digested in ~10 ml of HCl at 85°C for 4 hours. After digestion, the sample was filtered using a sand core funnel (class F) and diluted to ~20 ml using HCl (2 wt %) for ICP-MS measurement. For total REE quantification, CFA raw materials (~30 mg) were digested overnight at 95°C in a mixture of concentrated HF (48 wt %, 2 ml) and concentrated HNO_3_ (15 M, 2 ml). The sample was then dried in an oven at 100°C and redigested overnight at 95°C in a mixture of concentrated HNO_3_ (15 M, 1 ml), H_2_O_2_ (30 to 32%, 1 ml), and ultrapure water (5 ml, high-performance liquid chromatography grade). After the redigestion, all the solids were dissolved, and the sample was diluted to 50 ml using ultrapure water for ICP-MS measurement.

For the BR samples, HNO_3_-extractable REE contents were measured. Samples (~50 mg; BR raw materials or the activated BR by FJH) were digested using HNO_3_ (0.5 M) at room temperature for 24 hours. The sample was filtered using a sand core funnel (class F) for ICP-MS measurement.

For the e-waste samples, HCl-extractable REE contents were measured. Samples (~50 mg; e-waste raw materials or the activated e-waste by FJH) were digested using 10 ml of HCl (1 M) at 85°C for 4 hours. The sample was then filtered using a sand core funnel and diluted to 20 ml using ultrapure water for ICP-MS measurement.

For the CB as control, HCl-extractable REE contents were measured. Samples (~50 mg) were digested using 10 ml of HCl (1 M) at 85°C for 4 hours. The sample was then filtered using a sand core funnel and diluted to 20 ml using ultrapure water for ICP-MS measurement.

The ICP-MS measurement was conducted using a PerkinElmer Nexion 300 ICP-MS system. In the measurement, the primary REE interferences were oxides of lighter elements. To avoid the introduction of notable error, the formation of oxides was monitored using a mass ratio of 156:140 (CeO/Ce), which was kept below 3%. The detection limits for REE are in the level of 0.5 to 5 parts per trillion (ppt). The REE mixture standard was used (MilliporeSigma; periodic table mix 3 for ICP; 16 elements; 10 mg liter^−1^ each; Sc, Y, La, Ce, Pr, Nd, Sm, Eu, Gd, Tb, Dy, Ho, Er, Tm, Yb, and Lu in 5 wt % nitric acid). In our analyses, the standards were in the concentration range of 1 to 1000 parts per billion. All the analyzed samples were carefully diluted into the concentration range, which is at least two orders of magnitude higher than the detection limit of quantification. All the samples were measured three times to afford the SDs.

The impurities, including Al, Si, Fe, Mg, Zn, Co, Ni, Cr, and Cu, in the leachates were also measured using the ICP-MS. The mixture standard was used (MilliporeSigma, periodic table mix 1 for ICP; 33 elements; 10 mg liter^−1^ each; Al, As, Ba, Be, Bi, B, Ca, Cd, Cs, Cr, Co, Cu, Ga, In, Fe, Pb, Li, Mg, Mn, Ni, P, K, Rb, Se, Si, Ag, Na, Sr, S, Te, Tl, V, and Zn in 10 wt % nitric acid containing HF traces). ICP-MS is difficult to be used to define Ca due to the primary isotope of argon, which is at *m*/*z* (mass/charge ratio) = 40. Hence, ICP optical emission spectrometry (ICP-OES) was used to quantify Ca. The ICP-OES measurement was conducted using a PerkinElmer Optima 8300 ICP-OES.

### Characterization

SEM images were obtained using a FEI Quanta 400 ESEM field emission microscope at 10 kV. XPS spectra were collected using a PHI Quantera XPS system under the pressure of 5 × 10^−9^ torr. Elemental XPS spectra were collected using a step size of 0.1 eV and a pass energy of 26 eV. The XPS spectra were calibrated using the standard C 1*s* peak at 284.8 eV. XRD patterns were obtained using a Rigaku D/Max Ultima II system with a Cu Kα radiation (λ = 1.5406 Å). TGA was conducted in air at a heating rate of 10°C min^−1^ to 1000°C using a Q-600 Simultaneous TGA/DSC (differential scanning calorimetry) from TA Instruments. The temperature measurement was conducted using an infrared thermometer (Micro-Epsilon) with a temperature measurement range of 1000° to 3000°C and a time resolution of 1 ms. The optical image of the sample during FJH was captured using an ultrafast camera (Chronos 1.4). The color image was converted into an intensity matrix using MATLAB based on which the temperature map was obtained by fitting the Stefan-Boltzmann law

$$j=\sigma T^{4}$$ (1)

where *j* is the blackbody radiant emittance, σ is a constant of proportionality, and *T* is the thermodynamic temperature.

### Calculation

The dissolution curves are calculated on the basis of Visual MINTEQ 3.1. Extra Cl^−^ is added to compensate for the charge balance. The solubility of the REE oxide is estimated on the basis of the related REE hydroxide [e.g., Y(OH)_3_ was used to estimate the dissolution of Y_2_O_3_] due to the lack of *K*_sp_ data of REE oxides. Nevertheless, the calculated results of hydroxides match well with the ones calculated from the oxide counterparts in a previous report (*7*). The samples with mass of 1 g were dissolved in 100-ml solution to get the dissolution percentage.

## Supplementary Materials

### This PDF file includes:

Supplementary Text 1 to 4 Fig. S1 to S20 Table S1 to S4 References

## References

[1] T. Cheisson, E. J. Schelter, Rare earth elements: Mendeleev’s bane, modern marvels. Science 363, 489–493 (2019). 30705185 10.1126/science.aau7628

[2] J. C. K. Lee, Z. G. Wen, Pathways for greening the supply of rare earth elements in China. Nat. Sustain. 1, 598–605 (2018).

[3] R. K. Jyothi, T. Thenepalli, J. W. Ahn, P. K. Parhi, K. W. Chung, J.-Y. Lee, Review of rare earth elements recovery from secondary resources for clean energy technologies: Grand opportunities to create wealth from waste. J. Clean. Prod. 267, 122048–122073 (2020).

[4] R. K. Taggart, J. C. Hower, G. S. Dwyer, H. Hsu-Kim, Trends in the rare earth element content of U.S.-based coal combustion fly ashes. Environ. Sci. Technol. 50, 5919–5926 (2016). 27228215 10.1021/acs.est.6b00085

[5] R. C. Smith, R. K. Taggart, J. C. Hower, M. R. Wiesner, H. Hsu-Kim, Selective recovery of rare earth elements from coal fly ash leachates using liquid membrane processes. Environ. Sci. Technol. 53, 4490–4499 (2019). 30907587 10.1021/acs.est.9b00539

[6] W. Zhang, A. Noble, X. Yang, R. Honaker, A comprehensive review of rare earth elements recovery from coal-related materials. Minerals 10, 451–478 (2020).

[7] P. Liu, R. Huang, Y. Tang, Comprehensive understandings of rare earth element (REE) speciation in coal fly ashes and implication for REE extractability. Environ. Sci. Technol. 53, 5369–5377 (2019). 30912650 10.1021/acs.est.9b00005

[8] P. K. Sahoo, K. Kim, M. A. Powell, S. M. Equeenuddin, Recovery of metals and other beneficial products from coal fly ash: A sustainable approach for fly ash management. Int. J. Coal Sci. Technol. 3, 267–283 (2016).

[9] A. Middleton, D. M. Park, Y. Jiao, H. Hsu-Kim, Major element composition controls rare earth element solubility during leaching of coal fly ash and coal by-products. Int. J. Coal Geol. 227, 103532–103539 (2020).

[10] E. A. Deady, E. Mouchos, K. Goodenough, B. J. Williamson, F. Wall, A review of the potential for rare-earth element resources from European red muds: Examples from Seydişehir, Turkey and Parnassus-Giona, Greece. Mineral. Mag. 80, 43–61 (2016).

[11] R. M. Rivera, B. Ulenaers, G. Ounoughene, K. Binnemans, T. Van Gerven, Extraction of rare earths from bauxite residue (red mud) by dry digestion followed by water leaching. Miner. Eng. 119, 82–92 (2018).

[12] S. Reid, J. Tam, M. F. Yang, G. Azimi, Technospheric mining of rare earth elements from bauxite residue (red mud): Process optimization, kinetic investigation, and microwave pretreatment. Sci. Rep. 7, 15252 (2017). 29127406 10.1038/s41598-017-15457-8 PMC5681656

[13] S. Maroufi, R. K. Nekouei, R. Hossain, M. Assefi, V. Sahajwalla, Recovery of rare earth (i.e., La, Ce, Nd, and Pr) oxides from end-of-life Ni-MH battery via thermal isolation. ACS Sustain. Chem. Eng. 6, 11811–11818 (2018).

[14] V. G. Deshmane, S. Z. Islam, R. R. Bhave, Selective recovery of rare earth elements from a wide range of e-waste and process scalability of membrane solvent extraction. Environ. Sci. Technol. 54, 550–558 (2020). 31794204 10.1021/acs.est.9b05695

[15] S. Peelman, D. Kooijman, J. Sietsma, Y. Yang, Hydrometallurgical recovery of rare earth elements from mine tailings and WEEE. J. Sustain. Metall. 4, 367–377 (2018).

[16] J. F. King, R. K. Taggart, R. C. Smith, J. C. Hower, H. Hsu-Kim, Aqueous acid and alkaline extraction of rare earth elements from coal combustion ash. Int. J. Coal Geol. 195, 75–83 (2018).

[17] Z. Wang, S. Dai, J. Zou, D. French, I. T. Graham, Rare earth elements and yttrium in coal ash from the Luzhou power plant in Sichuan, Southwest China: Concentration, characterization and optimized extraction. Int. J. Coal Geol. 203, 1–14 (2019).

[18] R. K. Taggart, J. C. Hower, H. Hsu-Kim, Effects of roasting additives and leaching parameters on the extraction of rare earth elements from coal fly ash. Int. J. Coal Geol. 196, 106–114 (2018).

[19] S. Dou, J. Xu, X. Cui, W. Liu, Z. Zhang, Y. Deng, W. Hu, Y. Chen, High-temperature shock enabled nanomanufacturing for energy-related applications. Adv. Energy Mater. 10, 2001331–2001345 (2020).

[20] Y. G. Yao, Z. N. Huang, P. F. Xie, S. D. Lacey, R. J. Jacob, H. Xie, F. J. Chen, A. M. Nie, T. C. Pu, M. Rehwoldt, D. W. Yu, M. R. Zachariah, C. Wang, R. Shahbazian-Yassar, J. Li, L. B. Hu, Carbothermal shock synthesis of high-entropy-alloy nanoparticles. Science 359, 1489–1494 (2018). 29599236 10.1126/science.aan5412

[21] R. Jiang, Y. Da, X. Han, Y. Chen, Y. Deng, W. Hu, Ultrafast synthesis for functional nanomaterials. Cell Rep. Phys. Sci. 2, 100302 (2021).

[22] Y. Chen, G. C. Egan, J. Wan, S. Zhu, R. J. Jacob, W. Zhou, J. Dai, Y. Wang, V. A. Danner, Y. Yao, K. Fu, Y. Wang, W. Bao, T. Li, M. R. Zachariah, L. Hu, Ultra-fast self-assembly and stabilization of reactive nanoparticles in reduced graphene oxide films. Nat. Commun. 7, 12332 (2016). 27515900 10.1038/ncomms12332 PMC4990634

[23] C. Wang, W. Ping, Q. Bai, H. Cui, R. Hensleigh, R. Wang, A. H. Brozena, Z. Xu, J. Dai, Y. Pei, C. Zheng, G. Pastel, J. Gao, X. Wang, H. Wang, J.-C. Zhao, B. Yang, X. Zheng, J. Luo, Y. Mo, B. Dunn, L. Hu, A general method to synthesize and sinter bulk ceramics in seconds. Science 368, 521–526 (2020). 32355030 10.1126/science.aaz7681

[24] D. X. Luong, K. V. Bets, W. A. Algozeeb, M. G. Stanford, C. Kittrell, W. Chen, R. V. Salvatierra, M. Q. Ren, E. A. McHugh, P. A. Advincula, Z. Wang, M. Bhatt, H. Guo, V. Mancevski, R. Shahsavari, B. I. Yakobson, J. M. Tour, Gram-scale bottom-up flash graphene synthesis. Nature 577, 647–651 (2020). 31988511 10.1038/s41586-020-1938-0

[25] W. Chen, J. T. Li, Z. Wang, W. A. Algozeeb, D. X. Luong, C. Kittrell, E. A. McHugh, P. A. Advincula, K. M. Wyss, J. L. Beckham, M. G. Stanford, B. Jiang, J. M. Tour, Ultrafast and controllable phase evolution by flash Joule heating. ACS Nano 15, 11158–11167 (2021). 34138536 10.1021/acsnano.1c03536

[26] N. H. Barbhuiya, A. Kumar, A. Singh, M. K. Chandel, C. J. Arnusch, J. M. Tour, S. P. Singh, The Future of flash graphene for the sustainable management of solid waste. ACS Nano 15, 15461–15470 (2021). 34633174 10.1021/acsnano.1c07571

[27] W. A. Algozeeb, P. E. Savas, D. X. Luong, W. Chen, C. Kittrell, M. Bhat, R. Shahsavari, J. M. Tour, Flash graphene from plastic waste. ACS Nano 14, 15595–15604 (2020). 33119255 10.1021/acsnano.0c06328

[28] P. A. Advincula, D. X. Luong, W. Chen, S. Raghuraman, R. Shahsavari, J. M. Tour, Flash graphene from rubber waste. Carbon 178, 649–656 (2021).

[29] M. Y. Stuckman, C. L. Lopano, E. J. Granite, Distribution and speciation of rare earth elements in coal combustion by-products via synchrotron microscopy and spectroscopy. Int. J. Coal Geol. 195, 125–138 (2018).

[30] S. V. Ushakov, K. B. Helean, A. Navrotsky, L. A. Boatner, Thermochemistry of rare-earth orthophosphates. J. Mater. Res. 16, 2623–2633 (2001).

[31] Y. Hikichi, T. Nomura, Melting temperatures of monazite and xenotime. J. Am. Ceram. Soc. 70, C-252–C-253 (1987).

[32] A. Kolker, C. Scott, J. C. Hower, J. A. Vazquez, C. L. Lopano, S. F. Dai, Distribution of rare earth elements in coal combustion fly ash, determined by SHRIMP-RG ion microprobe. Int. J. Coal Geol. 184, 1–10 (2017).

[33] D. Smolka-Danielowska, Rare earth elements in fly ashes created during the coal burning process in certain coal-fired power plants operating in Poland - Upper Silesian Industrial Region. J. Environ. Radioact. 101, 965–968 (2010). 20713303 10.1016/j.jenvrad.2010.07.001

[34] S. Dai, L. Zhao, J. C. Hower, M. N. Johnston, W. Song, P. Wang, S. Zhang, Petrology, mineralogy, and chemistry of size-fractioned fly ash from the Jungar Power Plant, Inner Mongolia, China, with emphasis on the distribution of rare earth elements. Energy Fuel 28, 1502–1514 (2014).

[35] D. Barreca, G. A. Battiston, D. Berto, R. Gerbasi, E. Tondello, Y_2_O_3_ thin films characterized by XPS. Surf. Sci. Spectra 8, 234–239 (2001).

[36] K. M. Cole, D. W. Kirk, S. J. Thorpe, Surface Y_2_O_3_ layer formed on air exposed Y powder characterized by XPS. Surf. Sci. Spectra 27, 024010–024020 (2020).

[37] A. M. de Asha, R. M. Nix, Oxidation of lanthanum overlay ers on Cu(111). Surf. Sci. 322, 41–50 (1995).

[38] J. P. H. Li, X. Zhou, Y. Pang, L. Zhu, E. I. Vovk, L. Cong, A. P. van Bavel, S. Li, Y. Yang, Understanding of binding energy calibration in XPS of lanthanum oxide by in situ treatment. Phys. Chem. Chem. Phys. 21, 22351–22358 (2019). 31576882 10.1039/c9cp04187g

[39] N. N. Greenwood, A. Earnshaw, *Chemistry of the Elements* (Butterworth-Heinemann, ed. 2, 1997), 299 pp.

[40] R. F. Service, Red alert. Science 369, 910–911 (2020). 32820108 10.1126/science.369.6506.910

[41] M. OchsenkuhnPetropulu, T. Lyberopulu, K. M. Ochsenkuhn, G. Parissakis, Recovery of lanthanides and yttrium from red mud by selective leaching. Anal. Chim. Acta 319, 249–254 (1996).

[42] M. Boni, G. Rollinson, N. Mondillo, G. Balassone, L. Santoro, Quantitative mineralogical characterization of karst bauxite deposits in the southern Apennines, Italy. Econ. Geol. 108, 813–833 (2013).

[43] X. Zeng, J. A. Mathews, J. Li, Urban mining of e-waste is becoming more cost-effective than virgin mining. Environ. Sci. Technol. 52, 4835–4841 (2018). 29616548 10.1021/acs.est.7b04909

[44] M. A. Alam, L. Zuga, M. G. Pecht, Economics of rare earth elements in ceramic capacitors. Ceram. Int. 38, 6091–6098 (2012).

[45] D. Bauer, D. Diamond, J. Li, D. Sandalow, P. Telleen, B. Wanner, *Critical Materials Strategy* (U.S. Department of Energy, 2010).

[46] V. V. Seredin, S. Dai, Coal deposits as potential alternative sources for lanthanides and yttrium. Int. J. Coal Geol. 94, 67–93 (2012).

[47] T. Wen, Q. Zhang, C. Guo, X. Liu, L. Pang, J. Zhao, Y. Yin, W. Shi, W. Chen, X. Tan, 3-MV compact very fast transient overvoltage generator for testing ultra-high-voltage gas-insulated switchgear. IEEE Electr. Insul. Mag. 30, 26–33 (2014).

[48] W. Chen, X. Cui, Foreword for the special section on AC and DC ultra high voltage technologies. CSEE J. Power Energy Syst. 1, 1–2 (2015).

[49] S. Peelman, Z. H. I. Sun, J. Sietsma, Y. Yang, in *Rare Earths Industry,* I. Borges De Lima, W. Leal Filho, Eds. (Elsevier, 2016), pp. 319–334.

[50] R. Kim, H. Cho, K. N. Han, K. Kim, M. Mun, Optimization of acid leaching of rare-earth elements from Mongolian apatite-based Ore. Minerals 6, 63 (2016).

[51] F. Xie, T. A. Zhang, D. Dreisinger, F. Doyle, A critical review on solvent extraction of rare earths from aqueous solutions. Miner. Eng. 56, 10–28 (2014).

[52] M. T. Schatzmann, M. L. Mecartney, P. E. D. Morgan, Synthesis of monoclinic monazite, LaPO_4_, by direct precipitation. J. Mater. Chem. 19, 5720–5722 (2009).

[53] Current prices of rare earths; https://en.institut-seltene-erden.de/aktuelle-preise-von-seltenen-erden/.

[54] W. D. Judge, G. Azimi, Recent progress in impurity removal during rare earth element processing: A review. Hydrometallurgy 196, 105435–105474 (2020).

[55] W. Zhang, X. Xie, X. Tong, Y. Du, Q. Song, D. Feng, Study on the effect and mechanism of impurity aluminum on the solvent extraction of rare earth elements (Nd, Pr, La) by P204-P350 in chloride solution. Minerals 11, 61–73 (2021).

[56] Q. Ye, G. Li, B. Deng, J. Luo, M. Rao, Z. Peng, Y. Zhang, T. Jiang, Solvent extraction behavior of metal ions and selective separation Sc^3+^ in phosphoric acid medium using P204. Sep. Purif. Technol. 209, 175–181 (2019).

[57] R. G. Silva, C. A. Morais, E. D. Oliveira, Selective precipitation of rare earth from non-purified and purified sulfate liquors using sodium sulfate and disodium hydrogen phosphate. Miner. Eng. 134, 402–416 (2019).

[58] F. Li, A. Gong, L. Qiu, W. Zhang, J. Li, Z. Liu, Diglycolamide-grafted Fe_3_O_4_/polydopamine nanomaterial as a novel magnetic adsorbent for preconcentration of rare earth elements in water samples prior to inductively coupled plasma optical emission spectrometry determination. Chem. Eng. J. 361, 1098–1109 (2019).

[59] Z. Lou, X. Xiao, M. Huang, Y. Wang, Z. Xing, Y. Xiong, Acrylic acid-functionalized metal–organic frameworks for Sc(III) selective adsorption. ACS Appl. Mater. Inter. 11, 11772–11781 (2019). 10.1021/acsami.9b00476 30852887

[60] C. Brabant, A. Khodakov, A. Griboval-Constant, Promotion of lanthanum-supported cobalt-based catalysts for the Fischer-Tropsch reaction. C. R. Chim. 20, 40–46 (2017).

[61] L. Schlapbach, H. R. Scherrer, XPS core level and valence band spectra of LaH_3_. Solid State Commun. 41, 893–897 (1982).

[62] A. Shelyug, A. Mesbah, S. Szenknect, N. Clavier, N. Dacheux, A. Navrotsky, Thermodynamics and stability of rhabdophanes, hydrated rare earth phosphates REPO_4_ · n H_2_O. Front. Chem. 6, 604 (2018). 30619814 10.3389/fchem.2018.00604 PMC6304437

[63] D. R. Lide, *CRC Handbook of Chemistry and Physics* (CRC Press, 2005); www.hbcpnetbase.com.

[64] D. D. Wagman, W. H. Evans, V. B. Schumm, I. Halow, *The NBS Tables of Chemical Thermodynamic Properties. Selected Values for Inorganic and C1 and C2 Organic Substances in SI Units* (American Chemical Society, 1982).

[65] Y. Zhang, I.-H. Jung, Critical evaluation of thermodynamic properties of rare earth sesquioxides (RE = La, Ce, Pr, Nd, Pm, Sm, Eu, Gd, Tb, Dy, Ho, Er, Tm, Yb, Lu, Sc and Y). Calphad 58, 169–203 (2017).

[66] S. A. Wood, I. M. Samson, The aqueous geochemistry of gallium, germanium, indium and scandium. Ore Geol. Rev. 28, 57–102 (2006).

[67] Z. S. Cetiner, S. A. Wood, C. H. Gammons, The aqueous geochemistry of the rare earth elements. Part XIV. The solubility of rare earth element phosphates from 23 to 150 degrees C. Chem. Geol. 217, 147–169 (2005).

