# Supplementary material for: Rare earth elements from waste
Source: Sci Adv. 2022 Feb 9;8(6):eabm3132. doi: 10.1126/sciadv.abm3132 (PMC8827657; doi:10.1126/sciadv.abm3132)
Supplement: Supplementary file 1 — Supplementary Text 1 to 4 Fig. S1 to S20 Table S1 to S4 References [file sciadv.abm3132_sm.pdf]

Science Advances
AAAS

Supplementary Materials for

Rare earth elements from waste

Bing Deng, Xin Wang, Duy Xuan Luong, Robert A. Carter, Zhe Wang, Mason B. Tomson, James M. Tour*

*Corresponding author. Email: tour@rice.edu

Published 9 February 2022, *Sci. Adv.* **8**, eabm3132 (2022)
DOI: 10.1126/sciadv.abm3132

This PDF file includes:

Supplementary Text 1 to 4
Fig. S1 to S20
Table S1 to S4
References

Supplementary Text

Supplementary Text 1. Calculation of the recovery yield.

The total REE contents in CFA raw materials, c_{total}(CFA-Raw), was measured by total digestion using a HF:HNO_3 digestion method (see details in Materials and Methods, fig. S6). The acid-leachable REE contents in CFA raw materials, c_0(CFA-Raw), were measured by HCl or HNO_3 leaching of the CFA raw materials. The REE recovery yield by acid leaching the CFA raw materials was calculated by Eq. S1,

$$Y_0 = \frac{c_0(\text{CFA−Raw})}{c_{\text{total}}(\text{CFA−Raw})} \qquad \text{(S1)}$$

The CFA raw materials and carbon black were mixed and underwent the FJH activation process. The obtained solid is termed as activated CFA. The acid-leachable REE content in the activated CFA, c(activated CFA), was measured by the same acid leaching procedure of CFA after FJH. The REE recovery yield by acid leaching the activated CFA was calculated by Eq. S2,

$$Y = \frac{c(\text{activated CFA})}{c_{\text{total}}(\text{CFA−Raw})} \qquad \text{(S2)}$$

Hence, the ratio of Y/Y_0 was calculated by Eq. S3,

$$\frac{Y}{Y_0} = \frac{c(\text{activated CFA})}{c_0(\text{CFA−Raw})} \qquad \text{(S3)}$$

The Y/Y_0 could be used as an index of the increase of the REE recovery yield from CFA by the FJH activation process. If $Y/Y_0 > 1$, the activation process has a positive effect and improves the recovery yield. Similarly, for BR and e-waste, the increase of recovery yield is calculated by using Eq. S4 and Eq. S5:

$$\frac{Y}{Y_0} = \frac{c(\text{activated BR})}{c_0(\text{BR−Raw})} \qquad \text{(S4)}$$

$$\frac{Y}{Y_0} = \frac{c(\text{activated e−waste})}{c_0(\text{e−waste−Raw})} \qquad \text{(S5)}$$

Supplementary Text 2. Strategy for scaling up the FJH process.

Joule heating is widely used in many devices and industrial processes, such as electric heaters and tube furnace. Flash Joule heating (FJH) reported here, is intrinsically a Joule heating process. For traditional Joule heating process, a constant and long-time alternating current (AC) or direct current (DC) is used as the electrical resources. In contrast, in the FJH process, which is firstly invented by our group for the synthesis of turbostratic graphene (*24*), a pulsed DC provided by the discharging of capacitors is used, which provides a much higher temperature in a short duration. The FJH process is scalable. We here conduct theoretical analysis of the FJH process to identify the key parameters determining the temperature; then, we experimentally demonstrate the scaling-up of the FJH process; we also provide conceptual prototype of the continuous production; lastly, we briefly mention the ongoing industrial scale application of the FJH process on graphene synthesis, which can be easily shifted for the REE recovery purpose.

Theoretical analysis of the scaling rule.

For scaling up, it is critical to maintain a constant temperature value and temperature distribution when the sample mass is increased.

The heat amount (Q) produced by Joule heating is calculated by Eq. S6,

$$Q = I^2Rt \qquad \text{(S6)}$$

where I is the current passing through the sample, R is the sample resistance, and t is the time. Considering heat amount per volume (Q_v), the equation could be revised to Eq. S7,

$$Q_v = j^2 \rho_e t \quad \text{(S7)}$$

where Q_v is the heat per volume, j is the current density, and ρ_e is the resistivity of the sample.

According to the heat transfer formula, the change of temperature could be calculated by Eq. S8,

$$Q = C_p m \Delta T \quad \text{(S8)}$$

where ΔT is the change of temperature, m is the mass of the sample, and C_p is the specific heat capacity. Since the specific capacity (C_p) is a constant for a specific type of sample, the change of temperature is determined by the heat amount.

Furthermore, we can revise the Eq. S8 per volume to Eq. S9,

$$Q_v = C_p \rho_m \Delta T \quad \text{(S9)}$$

where ρ_m is the density of the sample. Since the density (ρ_m) and the specific heat capacity (C_p) are constant for the sample, the change of temperature is linearly proportional to Q_v. In other word, regardless of the sample mass, the same temperature could be achieved as long as the Q_v remains the same.

Then, according to Eq. S7, since the resistivity (ρ_e) of the sample is constant for the sample, to keep a constant Q_v and t when scaling up the sample mass (m), we need to maintain a constant j, which is determined by Eq. S10,

$$j = \frac{I}{S} \quad \text{(S10)}$$

where I is the current passing through the sample, and S is the cross-sectional area. The current is calculated by Eq. S11,

$$I = \frac{q}{t} \quad \text{(S11)}$$

where q is the charge, and t is the time.

Supposing that the charges in the capacitor bank are discharged within the time of t, the charge could be calculated by Eq. S12,

$$q = CV \quad \text{(S12)}$$

where C is the total capacitance of the capacitor bank, and V is the charging voltage.

According to Eq. S10 to 12, the current density is determined by Eq. S13,

$$j = \frac{CV}{St} \quad \text{(S13)}$$

The sample is usually cylinder-shaped since we always use a quartz tube, so the sample mass is calculated by Eq. S14,

$$m = \rho_m S L \quad \text{(S14)}$$

where S is the cross-sectional area, and L is the sample length.

According to Eq. S13 and S14, the current density is determined by Eq. S15

$$j = \frac{CV\rho_m L}{mt} \quad \text{(S15)}$$

The density of the sample (ρ_m) is constant, and we can change the sample cross-sectional area to maintain a constant sample length of L. Hence, to maintain a constant j and t when the mass (m) of the sample is increased, there are two approaches: (1) to increase the Joule heating voltage V; and/or (2) to increase the capacitance of C.

<u>Scaling up demonstration.</u>

We here demonstrated the scaling up of the FJH process by using the first strategy, increasing the voltage. In most of the experiments (table S1) with the sample mass of m_0 = 0.2 g, we use a FJH voltage of V_0 = 120 V and capacitance of C_0 = 60 mF, and a cylinder-shape sample

with diameter of D_0 = 8 mm (S_0 ~ 50 mm^2). To scale up the reaction to m_1 = 2 g, we used a large sample size with diameter of D_1 = 16 mm (S_1 ~ 200 mm^2). According to Eq. S14 to S15, the voltage for the large-scale sample should be $V_1 = 2.5 \times V_0$ = 300 V. We used CFA-C as an example and demonstrated the FJH process with mass up to 2 g (fig. S16A). The reaction conditions are: m_1 = 2 g, D_1 = 16 mm, V_1 = 300 V, and t = 1 s. The result is shown in fig. S16B. The CFA-C is successfully activated with the increase of REE recovery yields to 150% to 190%, comparable to the results of the sample with smaller mass (Fig. 2H).

We can also scale up the FJH process by increasing the capacitance (C). In our first generation FJH system, which is used in this work, the capacitor bank is composed of 10 commercial aluminum electrolytic capacitor and has a total capacitance of C_0 = 60 mF. We also built a second generation FJH system in our lab with the total capacitance of C_2 = 0.624 F. By using a FJH voltage of V_2 =380 V and capacitance of C_2 = 0.624 F, we realized the sample mass of m_2 = 5 g per batch. Actually, our lab has already scaled the FJH process for the conversion of coal to graphene to ~17.6 kg day^{-1}. Since the main FJH process for graphene synthesis and for the REE recovery is pretty the same, it is safe to presume that the FJH process for REE recovery has the production rate of >10 kg day^{-1} in our research lab.

The conceptual prototype of the continuous production.

In our present work, the FJH process is done batch-by-batch. We presume that the FJH process could be done automatically in a continuous manner as we have demonstrated in our graphene production process. We provide here a conceptual prototype of the continuous production reactor (fig. S17). The continuous production process consists of four steps. The mixture of CFA/CB feedstock is loaded onto the chamber on the conveyor belt. The sample is compressed to a specific resistance. The sample then undergoes the FJH reaction. Lastly, the activated CFA product is collected. This is only one possible design. Considering the various commercially available continuous production processes and equipment, such as rolling-belt processes, our FJH process could be integrated into them for the purpose of REE recovery.

The commercial scaling up of the FJH process is ongoing.

The application of the FJH process to an industrial scale for the production of graphene is ongoing by Universal Matter Inc. (https://www.universalmatter.com/), with the targeted production rate of 1 ton day^{-1} by Q2 2022, and further scaling to 100 tons per day by 2023. To date, the production rate doubles every 9 weeks. The equipment and process designed and optimized for graphene production could be applied for REE recovery purpose. At the industrial scale, alternative current (AC) is a more feasible electrical resource than the direct current (DC). According to our above analysis, optimizing the voltage is one approach to scale up the FJH process. In industry, the high voltage or even ultrahigh voltage up to hundreds of kV are mature technologies, which could be applied in the REE process. Hence, the FJH process has a tried route to scalability for the REE recovery. The ongoing commercial scaling of the FJH process paves the way for future REE recovery from large-scale waste products.

Supplementary Text 3. Energy consumption calculation and profit estimation.

The electrical energy (E) consumption is calculated by Eq. S16,

$$E = \frac{(V_1^2 - V_2^2) \times C}{2 \times M} \quad \text{(S16)}$$

where V_1 and V_2 are the start voltage and the end voltage after the FJH, respectively, C is the capacitance (60 mF), and M is the mass per batch.

In a typical experiment with V_1 = 120 V, V_2 = 0 V, C = 60 mF, and M = 0.2 g, the energy is calculated to be,

E = 2.16 kJ g^{-1} = 6.0 × 10^{-4} kWh g^{-1} = 600 kWh ton^{-1}

Considering that the industrial electricity price in Texas, USA is \$0.02 kWh^{-1}, the cost for activating 1 ton of CFA would be P(electricity) = 12 \$ ton^{-1}.

Compared with the REE recovery without the activation process, the increased recovered REE amount by the activation process could be calculated by Eq. S17,

$$m = M \times c \times \Delta Y \quad \text{(S17)}$$

where m is the mass of the REE, M is the mass of CFA raw materials, c is the extractable content of REE from CFA raw materials, and ΔY is the improved REE recovery yield after the thermal activation process.

For REE recovery from CFA, the Sc, and critical REE, including Nd, Eu, Dy, Er, and Tb, contribute to more than 80% of the values (*4*). Considering 1 ton of CFA-C, the improved recovered REE amount would be:

m(Sc) = 23 g, m(Nd) = 29 g, m(Eu) = 2 g, m(Dy) = 5 g, m(Er) = 3 g, and m(Tb) = 3 g

The price of these critical REE are Sc (P = \$5735 kg^{-1}), Nd (P = \$64 kg^{-1}), Eu (P = \$285 kg^{-1}), Dy (P = \$375 kg^{-1}), Er (P = \$30 kg^{-1}), and Tb (P = \$770 kg^{-1}) in July 2019 (*53*). The values of the individual REE would be:

P(Sc) = \$132, P(Nd) = \$1.8, P(Eu) = \$0.6, P(Dy) = \$1.9, P(Er) = \$0.1, and P(Tb) = \$2.3

The value added of the REEs with the thermal activation process would be:

P(REE) = \$139 ton^{-1}

Here, we presume that the REE separation cost is constant for the directly leaching process and the activation-leaching process. The profit percentage for the activation process is calculated by Eq. S18,

$$\text{Profit percentage} = \frac{P(\text{REE}) - P(\text{electricity})}{P(\text{electricity})} \quad \text{(S18)}$$

The profit percentage is calculated to be ~11. The profit margin could be larger considering the improved recovery of other REE.

Supplementary Text 4. The impurities in the leachate and the envision on impurity removal.

REE separation is generally classified as primary separation (the separation of REE from other impurity elements), and secondary separation (the separation of individual REE) (*54*). The presence of metal impurities in the REE-containing leachate affects the subsequent REE separation efficiency by methods such as solvent extraction and ion exchange (*51, 54, 55*). Hence, the impurities usually need to be removed prior to the REE separation (*54*). Here, the impurities in the leachate are analyzed, and the removal of metal impurities and selective separation of individual REE from the leachate are envisioned.

The impurities in the leachate.

The composition of ores and secondary wastes often differ significantly from one source to another, and hence the impurities in the leachate also vary significantly in types and contents (*54*). The major impurities include Al, Si, Fe, Ca, Mg, Zn, Co, Ni, Cr, Cu, *etc*. Impurities, and especially the ratio of impurity content to REE content, could affect REE recovery and precipitation efficiencies by co-precipitation with REE, consuming the reagents, emulsification, *etc*. (*54, 55*). Here, we measured the impurity contents in our REE leachates from different secondary resources.

For CFA-F, the major impurities include Al, Si, Fe, Ca (10 to 100 ppm), Mg (1 to 10 ppm), Zn, Ni, Cr (0.1 to 1 ppm), and Co (0.01 to 0.1 ppm) (fig. S18). We found that ratio of *c*(REE)/*c*(Impurity) for the activated CFA-F is larger than that of the CFA-F raw materials for all the tested pH conditions ranging from 0 to 2 (fig. S18D). This means that the FJH process helps to increase the REE leachability while to some extent reduce the impurity leachability from CFA-F, which is beneficial for impurity removal. For CFA-C, the major impurities include Al, Si (>100 ppm), Fe, Ca, Mg (10 to 100 ppm), Zn (1 to 10 ppm), Ni, Cr (0.1 to 1 ppm), and Co (0.01 to 0.1 ppm) (fig. S19). Similarly, the ratio of *c*(REE)/*c*(Impurity) for the activated CFA-C is larger than that of the CFA-C raw materials for the tested pH of 0 and 1 (fig. S19C).

For BR, the major impurities include Al, Fe (>100 ppm), Si, Ca (10 to 100 ppm), Mg, Ni, Cr, Cu (1 to 10 ppm), Zn (0.1 to 1 ppm), and Co (0.01 to 0.1 ppm) (fig. S20A). The ratio of *c*(REE)/*c*(Impurity) for the BR raw materials is larger than that of the activated BR (fig. S20B). This means that the FJH also enhances the impurity leachability, especially Fe (fig. S20A). For e-waste, the major impurities include Cu (>100 ppm), Al, Si, Fe, Zn (10 to 100 ppm), Ca, Mg, Ni (1 to 10 ppm), and Co and Cr (0.1 to 1 ppm) (fig. S20C). The ratio of *c*(REE)/*c*(Impurity) for the activated e-waste is larger than that of the e-waste raw materials (fig. S20D).

The above impurity analysis would be helpful for the design of appropriate downstream REE purification and separation processing. The impurities content and the ratio of impurities to REE could be further optimized by adjusting the leaching conditions (*e.g.*, pulp density, pH, acid type, temperature, time, *etc*.).

Discussion on possible removal of impurities from the leach liquors.

Many techniques, including solvent extraction, ion exchange or adsorption, and selective precipitation, have been widely used to remove the impurities in leach liquor (*54*). The applicable route significantly depends on the impurities type and content, and the target application of REE. In our leach liquor obtained from CFA or BR, the major impurities are Al, Si, Fe, Mg, and Ca with concentration >10 ppm, while Cu and Zn should also be considered for the leach liquor from e-waste. Many methods have already been widely used for removing these impurities (*54*). For example, for the Fe-containing solution, acidic extractants such as di-(2-ethylhexyl)phosphoric

acid (D2EHPA) can selectively extract REE with appropriate extractant concentration and organic/aqueous ratio (*56*). For Al impurity, a significant amount can be removed through selective precipitation by adjusting the pH of the leach liquor (*57*). Ca and Mg impurities usually do not co-extract with REE with the upper tolerance limit being 1500 ppm (*58*), which is much higher than the contents in our leach liquor (fig. S18 to 20). Thus, Ca and Mg are not particularly problematic in REE extraction. For Cu and Zn, they do not usually co-extract with REE during cation solvent extraction or ion exchange (*58, 59*).

Discussion on the REE separation by solvent extraction.

Nowadays, solvent extraction is the most appropriate commercial technology for REE separation. The separation is generally done by primary separation and secondary separation. For the primary separation, D2EHPA is usually used to concentrate the REE from dilute solutions because of the high distribution coefficients. Subsequently, cation exchangers, solvation extractants, and anion exchangers are used to separate individual REE. Up to hundreds of stages of mixer and settler may be assembled to achieve the necessary separation and purity of REE, which have already been the routine scheme in many commercial REE extraction plants (*51*).

Our REE-containing leachates could undergo similar procedures to get the individual REE. Moreover, the extraction of REE with cation exchangers is promoted by increasing the aqueous phase pH (*51*). Here, we realized the high REE leachability at a relatively high pH (*e.g.*, pH 2 for CFA-F, and pH 1 for CFA-C), so the as-obtained leach liquors already have high pH. This could reduce the use of chemical agents to neutralize the leachate and be beneficial for the subsequent REE separation. Hence, existing separations technologies can be exploited to work with the REE extracts obtained through FJH. The mixtures obtained by FJH are often less cumbersome than those generated through the mining of ores, which represent another major benefit of the recycling scheme.

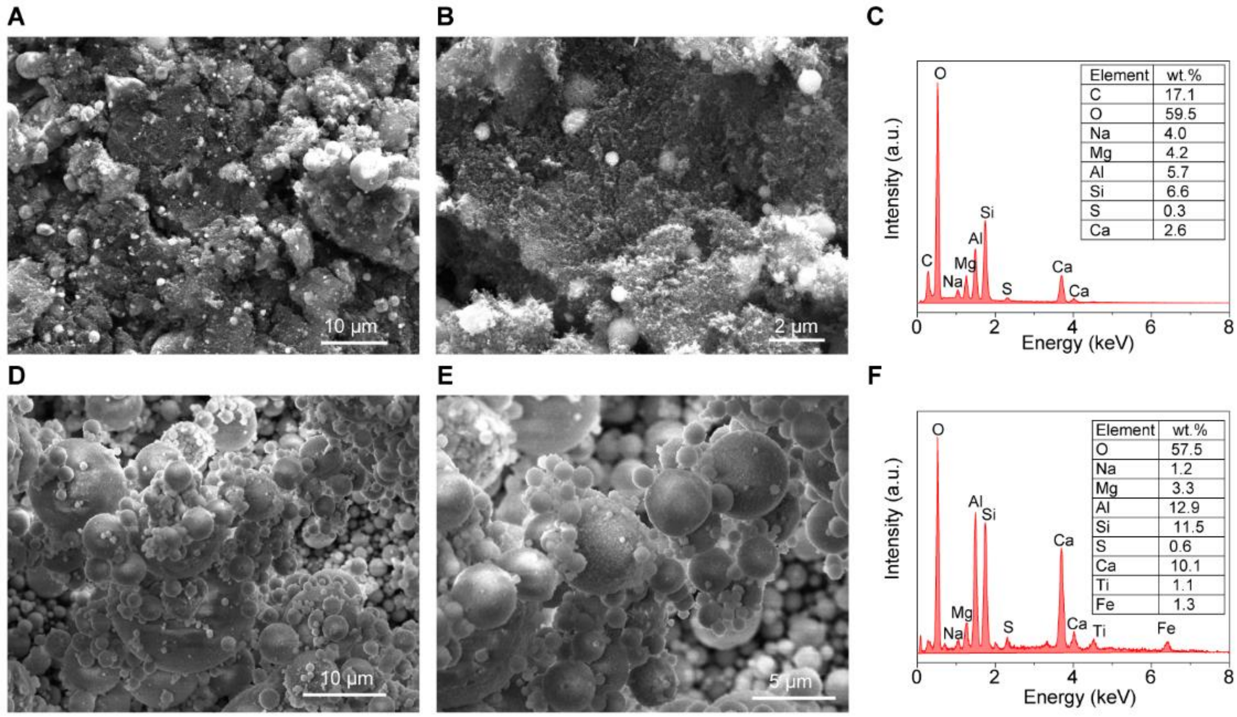

Fig. S1. Morphology and composition characterization of CFA by SEM and EDS. (**A** to **B**) SEM images of CFA-F. (**C**) EDS spectrum of CFA-F. Inset, the weight percentage of major elements in CFA-F. (**D** to **E**) SEM images of CFA-C. (**F**) EDS spectrum of CFA-C. Inset, the weight percentage of major elements in CFA-C.

The SEM images show that the CFA-C is composed of spherical particles with size of a few to tens of μm. In contrast, the size of CFA-F is smaller. The major elements in CFA-F are C, O, Na, Mg, Al, Si, S, and Ca. The major elements in CFA-C are O, Na, Mg, Al, Si, S, Ca, Ti, and Fe. The CFA-F has a high content of C while CFA-C has negligible C content. In contrast, the Ca content in CFA-C is significantly higher than that in CFA-F. Previous report showed that a higher content of Ca contributes to the higher acid extractability of REE (*4*).

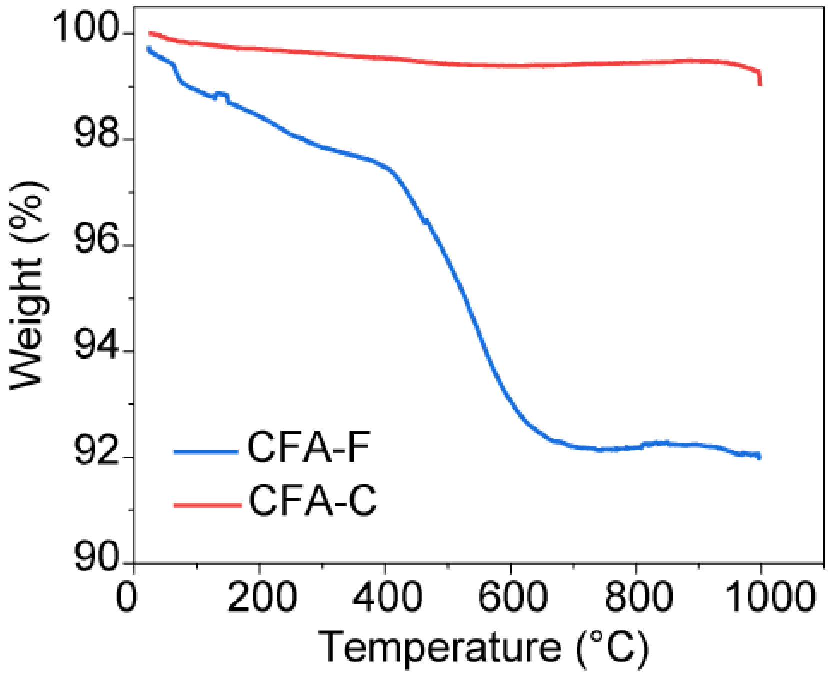

Fig. S2. TGA curve of CFA in air with a heating rate of 10 °C min^{-1}.

The TGA curve of CFA-C shows an obvious weight loss of ~8 wt% when the temperature rises to ~700 °C. This is probably caused by the combustion of C, since there is a high C content in CFA-F according to the EDS analysis (fig. S1). In contrast, the CFA-F only shows a minor weight loss up to 1000 °C. In CFA, the major components are metal oxides, such as CaO, Fe_2O_3, and SiO_2, which are stable in air up to 1000 °C.

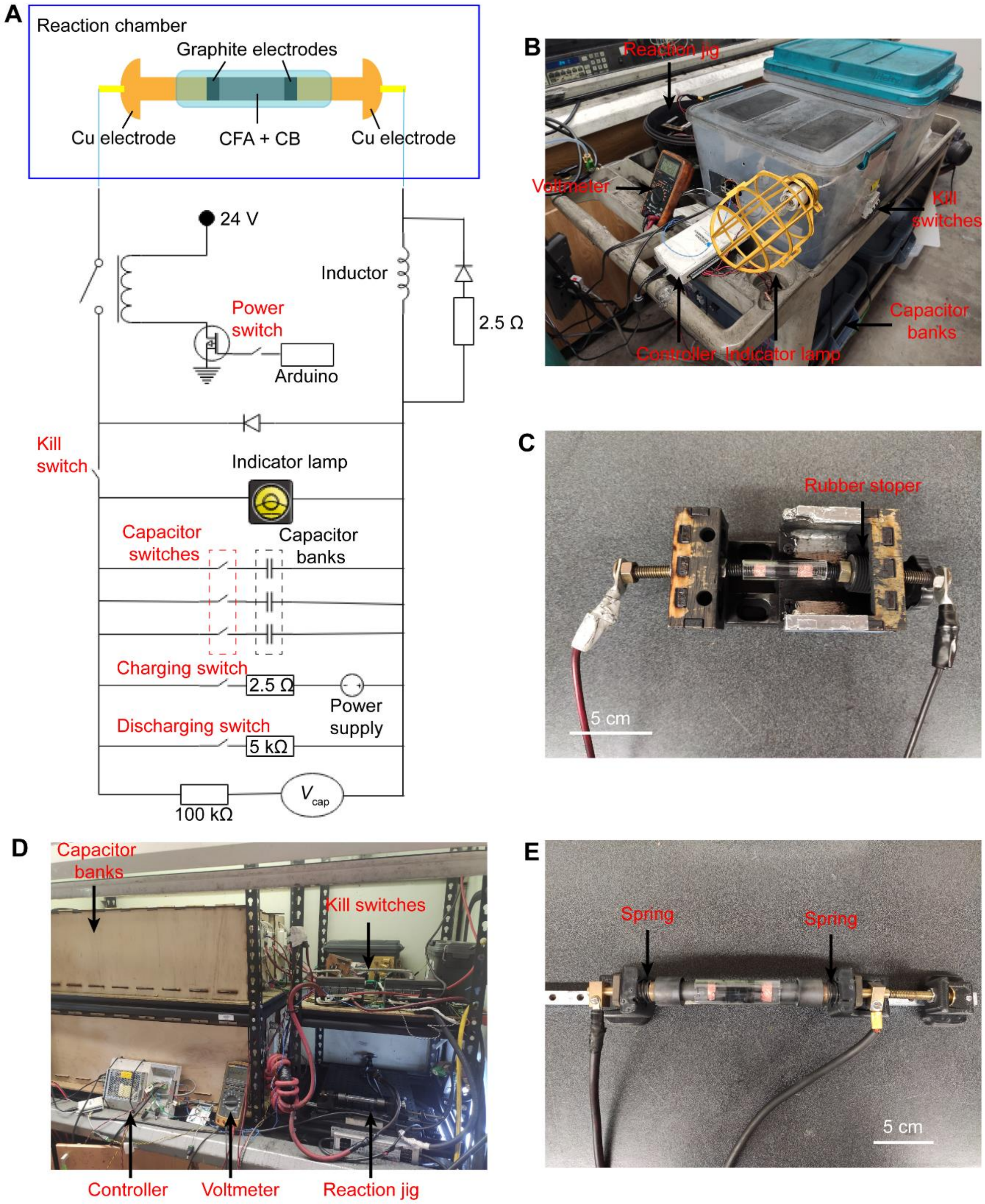

Fig. S3. The FJH system. (**A**) Schematic diagram of the FJH system. (**B**) The picture of the FJH system with the total capacitance of 0.06 F. (**C**) The smaller FJH jigs to connect the sample and the FJH system for 200-mg synthesis. (**D**) The picture of the larger FJH system with total capacitance of 0.624 F. (**E**) The larger FJH jigs to connect the sample and the FJH system for 2-g synthesis. The rubber stopper in (C) and the springs in (E) provide gradual compression to the sample during FJH.

Electrical components:

The details of the electrical components used in the FJH system could be found in our previous publication (*24*). We also listed major of them here.

1. Capacitors: Aluminum electrolytic capacitors (Mouser #80-PEH200YX460BQU2, 450 V, 6 mF). For the smaller FJH system, the capacitor bank is composed of 10 such capacitors with the total capacitance of 0.06 F. This capacitor bank is suitable for the reaction with batch size ≤ 0.5 g. For the larger FJH system, the capacitor bank is composed of 104 such capacitors with the total capacitance of 0.624 F. This capacitor bank is suitable for the reaction with batch size up to 10 g.
2. Mechanical relay: 900 V, 500 A (TE Connectivity LEV200A5ANA)
3. Power supply: LED Power Supplies 299.6 W 214–428 V 700 mA (Mouser #709-HLG320H-C700B).
4. V_{cap}: Multimeter Fluke 189
5. Charging and discharging switches: 400 V, 6 A breaker (ABB S 282 K 6 A)
6. Capacitor switches: 277 V, 10 A breaker (ABB S201P-C10)
7. Kill switch: 440 V, 630 A breaker (AAB S283 UC Z 63A)
8. Inductor: 24 mH (Mouser #553-C-80U)
9. Controller: Arduino Uno with LCD display
10. Power supply: LED Power Supplies 299.6 W 214-428 V 700 mA (Mouser #709-HLG320H-C700B)

Safety guidelines:

1. Enclose or carefully insulate the wire connections.
2. All connections and wires must be suitable for high voltages and currents.
3. Users should obey the one hand rule: use only one hand when working on the system, with the other hand not touching any grounded surface.
4. Keep in mind that the system can discharge thousands of Joules in milliseconds, which could cause components such as relays to explode.
5. Keep a voltmeter with high voltages test available at all times. When working on the capacitor banks, always check the voltage on each.
6. Wear thick rubber gloves that extending to the elbows when using the apparatus to protect from electrocution.
7. The reliability and robustness of the system should be confirmed by an experienced electrical technician.

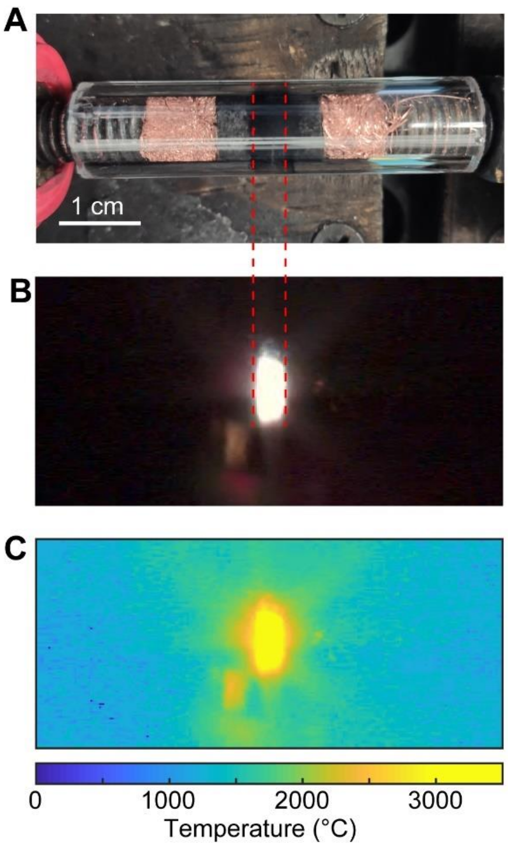

Fig. S4. Temperature map of the sample during FJH. (**A**) Optical image of the sample before FJH. (**B**) Optical image of the sample during FJH. (**C**) Temperature map of the sample during FJH. The dark regions on each side of the sample are graphite electrodes.

According to the Stefan-Boltzmann law, the blackbody radiant emittance (j) is proportional to the fourth power of the blackbody's thermodynamic temperature (T),

$$j = \sigma T^4 \qquad \text{(S19)}$$

where σ is a constant of proportionality. Inversely, the temperature of a sample could be evaluated based on its radiant intensity. Experimentally, we first captured the optical image of a sample during FJH process using an ultrafast camera (fig. S4B). Then, the color image was converted to a grayscale image, which was further converted to an intensity matrix using MATLAB. The highest temperature (T_{max} ~ 2970 °C) of the sample was obtained according to the IR thermometer measurement (Fig. 2C), which corresponds to the largest value (I_{max}) in the intensity matrix. Hence, the temperature (T) of each pixel of the image could be calculated using the intensity value (I) in the intensity matrix, based on the Stefan-Boltzmann law,

$$\frac{I}{I_{max}} = \left(\frac{T}{T_{max}}\right)^4 \qquad \text{(S20)}$$

The temperature distribution within the sample was plotted (fig. S4C). It is found that the temperature is very uniform throughout the entire sample without obvious gradient. The whole sample could achieve a high temperature of ~3000 °C, demonstrating that the FJH process has a homogenous heating capability.

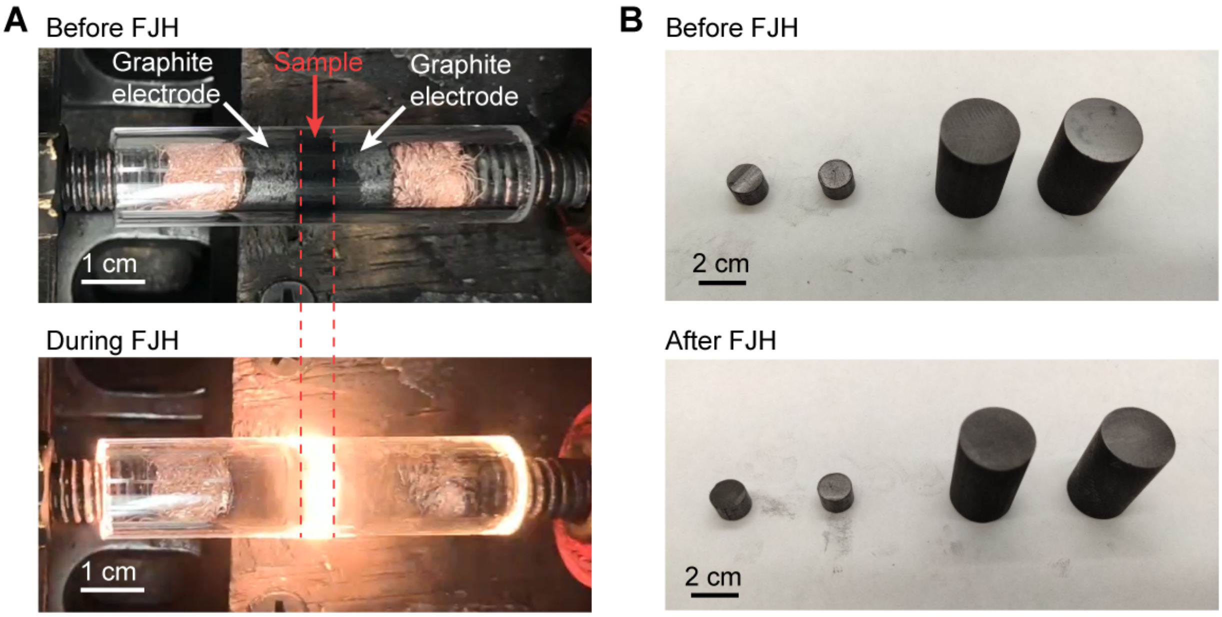

Fig. S5. Durability of the FJH system. (**A**) Optical images of the sample before (top) and during (bottom) the FJH. (**B**) Optical images of the graphite electrodes before (top) and after (bottom) the FJH.

The FJH setup has good durability. A high temperature (~3000 °C) could be achieved by the FJH process, but the high-temperature region is only limited to the sample. According to the Joule heating equation,

$$Q = I^2Rt \qquad \text{(S21)}$$

where I is the current passing through the sample, R is the resistance of the sample, and t is the discharging time, the electrical heat is proportional to the resistance. The graphite electrodes have much smaller resistance than the sample. Hence, the heat generated by the discharging process is imposed on the sample. As shown in fig. S5A, the strong light emission is limited on the sample region during the FJH process, while the graphite electrodes and other parts of the FJH system remain at relatively low temperatures. Graphite electrode has good thermal stability. In addition, the FJH time is very short, with the 3000 °C temperature in tens of milliseconds (Fig. 2C). The graphite rods show no obvious change after the FJH process (fig. S5B), and the resistance remains the same. Hence, the FJH process will not damage the electrodes. Other parts of the FJH system are commercial electrical components such as conductive wires and capacitors, which are far away from the high-temperature sample. The same FJH setup was used for hundreds of times for our experiments without degradation. Hence, we concluded that the FJH system has very good durability.

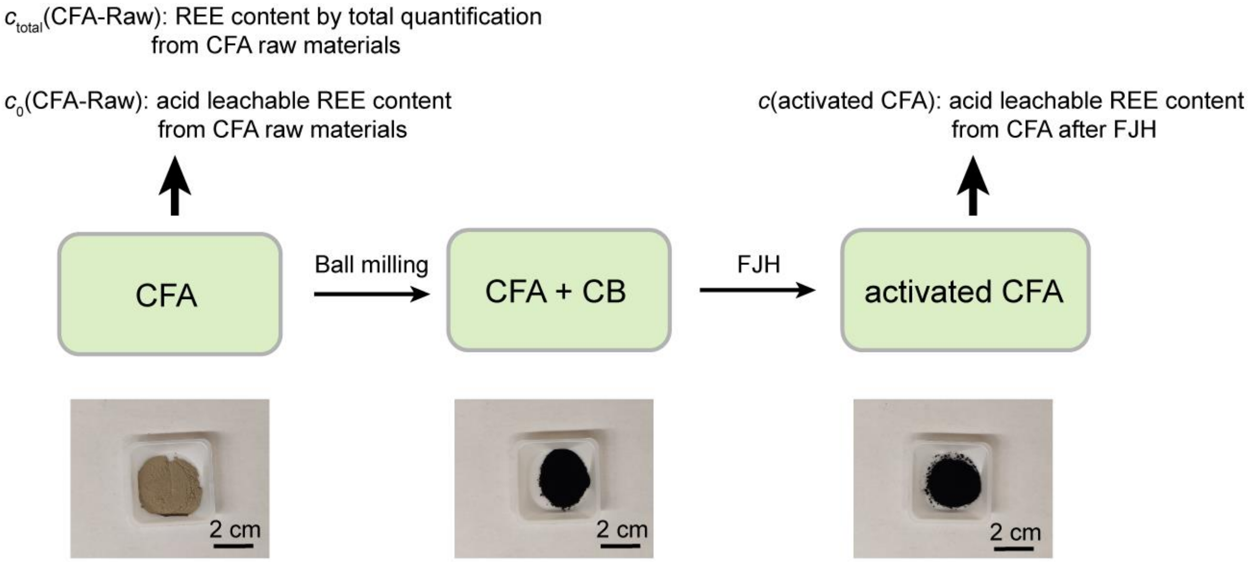

Fig. S6. Flow chart of REE recovery from secondary wastes by electrothermal activation. The total REE content in CFA raw materials, c_{total}(CFA-Raw), was measured by total digestion using a HF-HNO_3 digestion method (see details in Materials and Methods). The acid-leachable REE contents in CFA raw materials, c_0(CFA-Raw), were measured by HCl or HNO_3 leaching of the CFA raw materials. The CFA raw materials and carbon black were mixed and underwent the FJH activation process. The obtained solid is termed as activated CFA. The acid-leachable REE contents in activated CFA, c(activated CFA), were measured by the same acid leaching procedure of the activated CFA. Then, the REE recovery yield by acid leaching of the CFA raw materials, and the activated CFA were calculated (Supplementary Text 1).

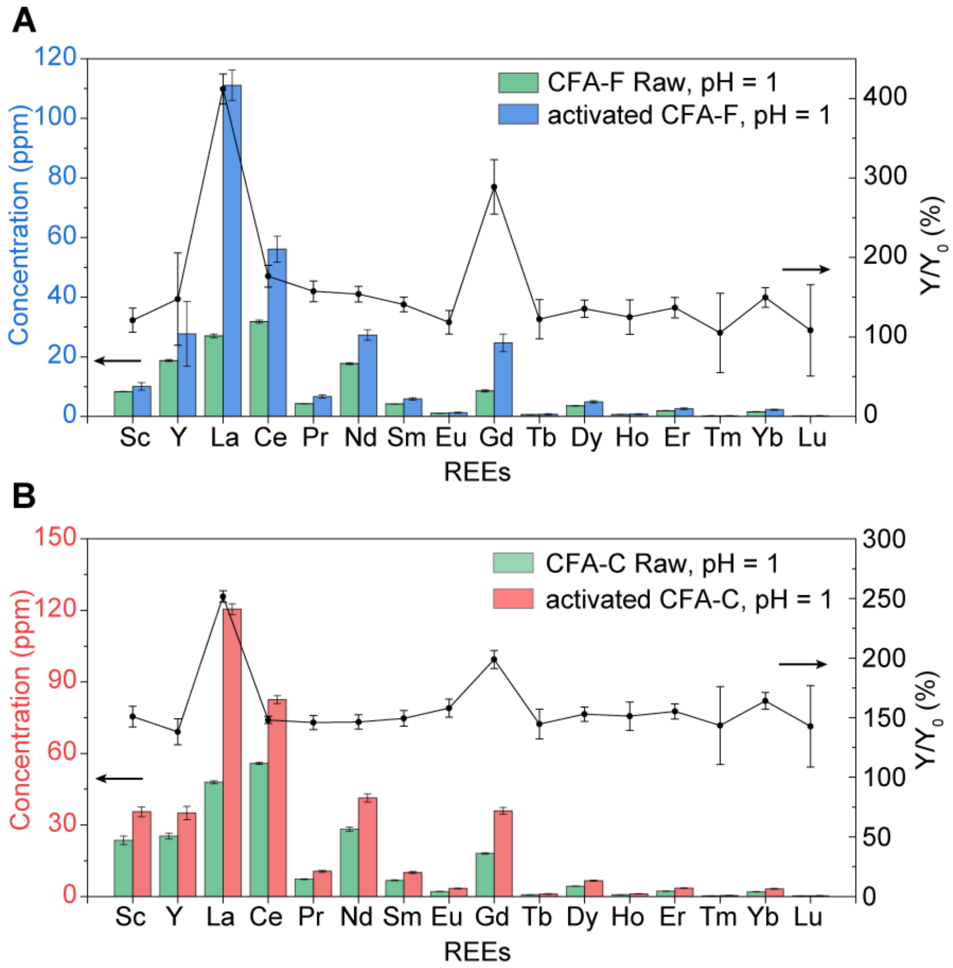

Fig. S7. REE leachability from CFA using 0.1 M HCl. (**A**) Acid-leachable REE contents (0.1 M HCl, 85 °C) from CFA-F raw materials and the activated CFA-F, and the increase of recovery yield. (**B**) Acid-leachable REE contents (0.1 M HCl, 85 °C) from CFA-C raw materials and the activated CFA-C, and the increase of recovery yield. Y_0 represents the REE recovery yield by 0.1 M HCl leaching the CFA raw materials, and Y represents the REE recovery yield by 0.1 M HCl leaching the activated CFA. For all the REE, the average values of Y/Y_0 were >100%, indicating that the FJH process increased the recovery yields. The error bars in A and B represent the standard derivation where N = 3.

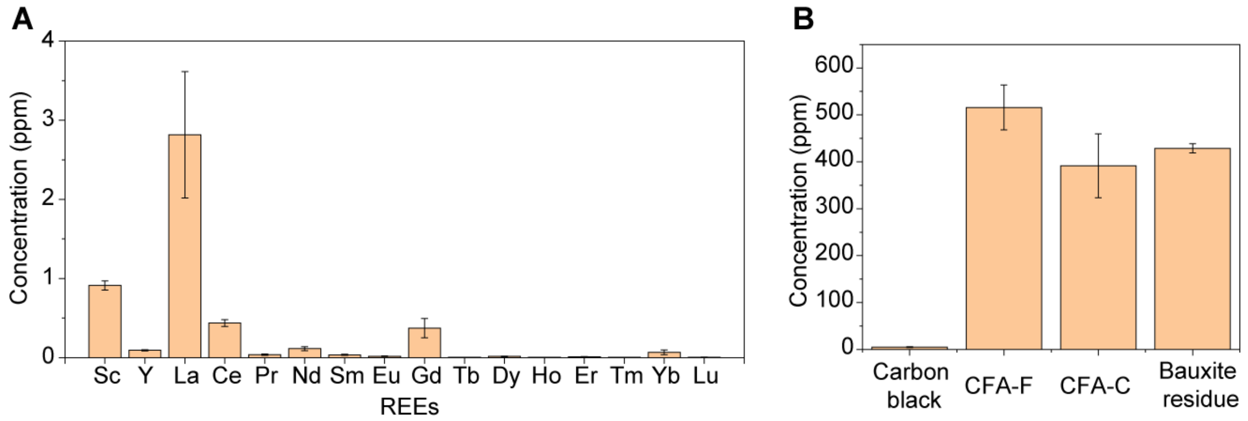

Fig. S8. REE contents in carbon black. (**A**) Individual REE content in carbon black. (**B**) Total REE content in carbon black, CFA-F, CFA-C, and bauxite residue (BR). All error bars in (A) and (B) represent the standard deviation where N = 3.

The REE contents in carbon black are low, ~1.0% of the REE contents in CFA-F, ~1.3% of the REE contents in CFA-C, and ~1.2% of the REE contents in BR. Hence, the carbon black additive will not induce significant error in our measurement.

In addition, carbon black is not the only choice as the conductive additive. In practical applications, the carbon black could be substituted with anthracite coal if desired, of any other inexpensive sources of mildly conductive carbon. In our cases, we used carbon black only since its REE content is low to exclude the error source induced by the conductive additive.

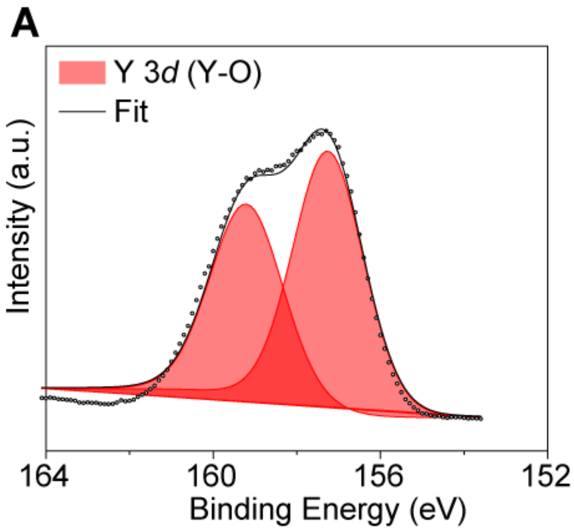

Fig. S9. XPS characterization of REE oxides. (**A**) XPS fine spectrum of Y in Y_2O_3 precursor. (**B**) XPS fine spectrum of La in La_2O_3 precursor.

The detailed peak fittings for La_2O_3, La_2O_3 after FJH, Y_2O_3, and Y_2O_3 after FJH are shown in table S4. The Y 3*d* was split into $3d_{5/2}$ and $3d_{3/2}$. In Y_2O_3, the peak positions for $3d_{5/2}$ and $3d_{3/2}$ are 157.4 eV and 159.4 eV, respectively, matching well with the literature reports (*35*, *36*). After FJH, the Y 3*d* were fitted by four peaks (Fig. 3E). The peaks at 157.5 eV and 159.6 eV are assigned to $3d_{5/2}$ and $3d_{3/2}$ of Y in Y_2O_3, respectively. The peaks at 156.4 eV and 158.5 eV are assigned to $3d_{5/2}$ and $3d_{3/2}$ of Y in Y metal, respectively, matching well with the literature report (*36*). The XPS analysis showed that the Y_2O_3 is reduced to Y metal during the FJH process, while the small ratio of Y_2O_3 might be from the surface oxidation.

For La_2O_3, the peaks at 834.8 eV and 838.2 eV correspond to $3d_{5/2}$ and its satellite peak (*38*, *60*). After FJH, in addition to the peaks from La_2O_3 at 834.8 eV and 838.1 eV (La $3d_{5/2}$ and its satellite peak), the peak fitting (Fig. 3F, table S4) shows major components at 836.0 eV and 839.6 eV, which are assigned to La $3d_{5/2}$ and it satellite peak from La metal, matching well with literature reports (*37*, *61*). The XPS analysis showed that La_2O_3 were reduced to La during the FJH process, while the small ratio of La_2O_3 might be from the surface oxidation.

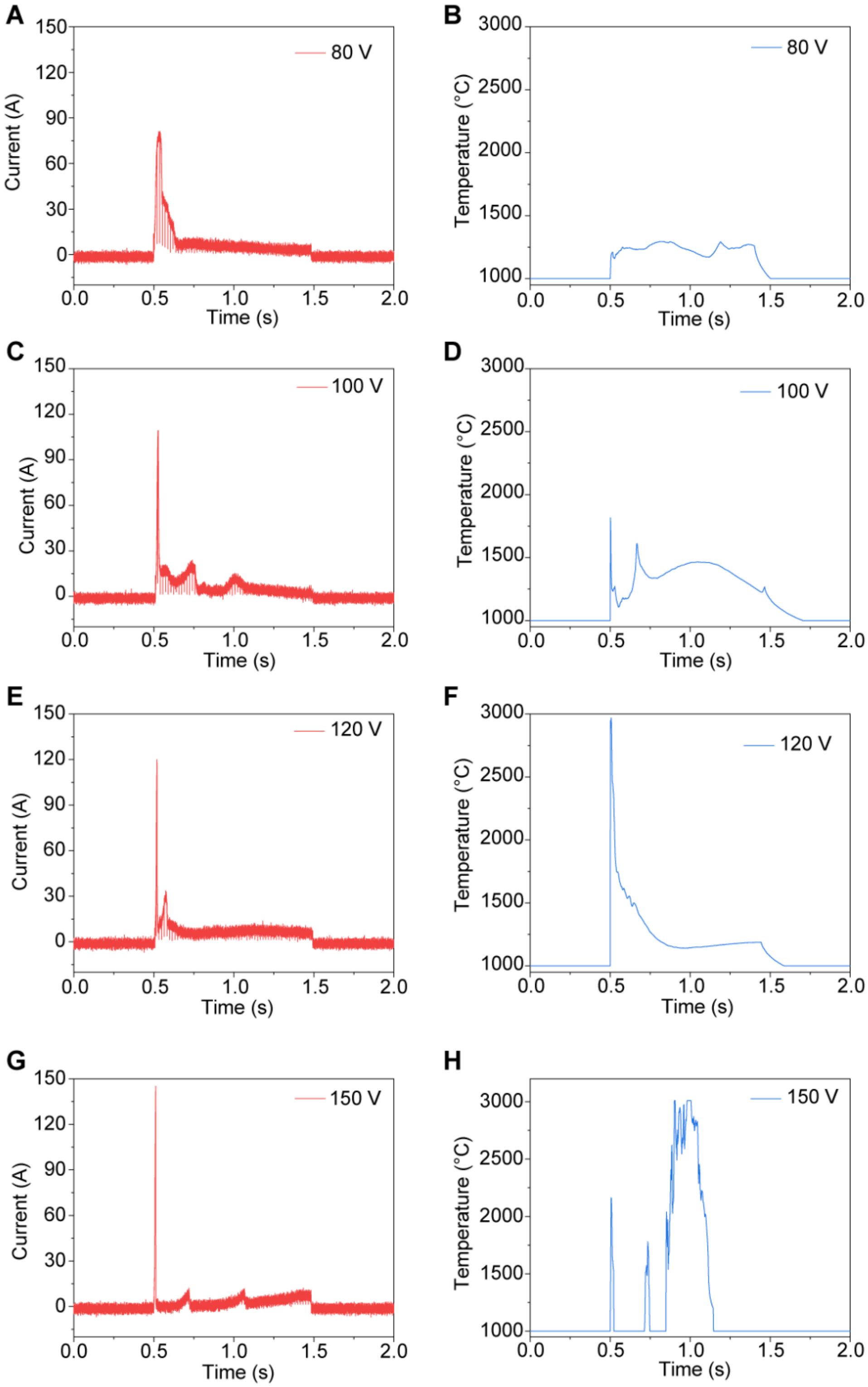

Fig. S10. Temperature measurement. Current curves and real-time temperature curves for (**A** to **B**) 80 V for 1 s, (**C** to **D**) 100 V for 1 s, (**E** to **F**) 120 V for 1 s, and (**G** to **H**) 150 V for 1 s.

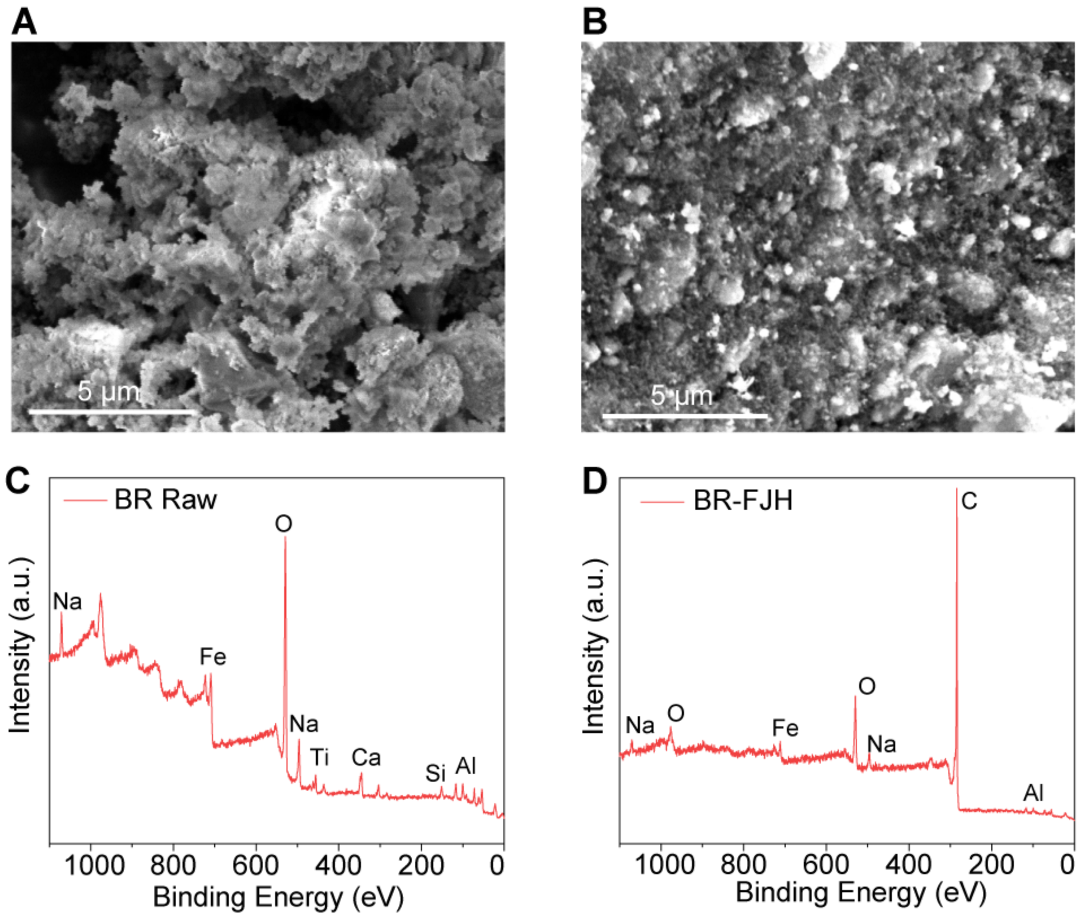

Fig. S11. Characterization of BR and BR after FJH. (**A**) SEM image of BR raw materials. (**B**) SEM image of BR after FJH. (**C**) XPS spectrum of BR raw materials. (**D**) XPS spectrum of BR after FJH.

After FJH, the particle size of the BR (fig. S11B) becomes smaller than the BR raw materials (fig. S11A), which would be helpful for the acid leaching. According to the XPS, after FJH, the O content in BR is significantly reduced, demonstrating the carbothermic reduction of the metal components in BR. This would also contribute to the acid leaching of REE for the same reasons we have explained for the recovery of REE from CFA in the main text (Fig. 3).

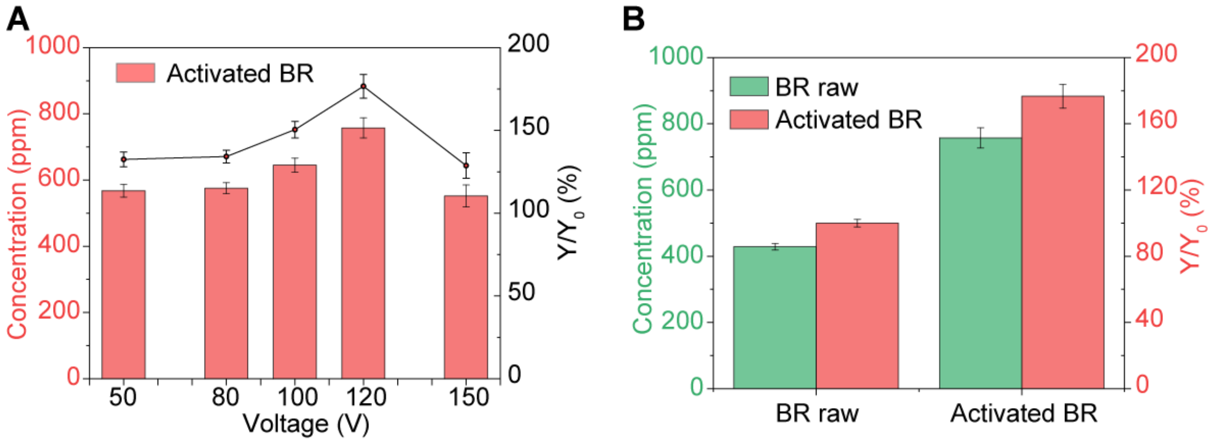

Fig. S12. FJH voltage dependent REE recovery yield from BR. (**A**) Acid leachable content of total REE (0.5 M HNO_3) from BR, and the increase of REE yield varied with FJH voltages. (**B**) The acid leachable REE content (0.5 M HNO_3), and the increase of recovery yield at 120 V FJH. Y_0 represents the REE recovery yield by directly leaching the BR raw materials. Y represents the REE recovery yield by leaching the activated BR. The error bar in A and B denotes the standard deviation where N = 3.

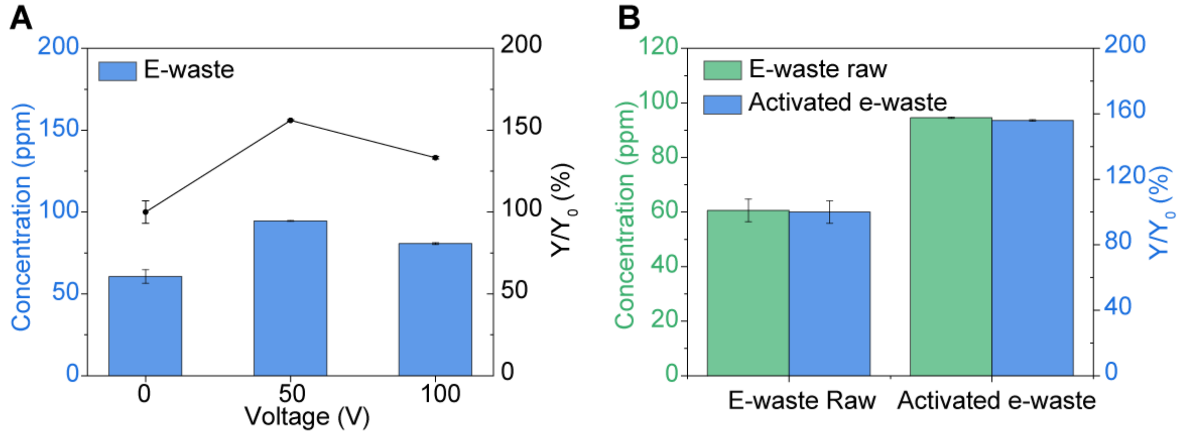

Fig. S13. Improving the REE recovery yield from e-waste by FJH activation. (**A**) Acid-leachable content of total REE (1 M HCl) from e-waste, and the increase of REE recovery yield varied with the FJH voltages. (**B**) The acid leachable content of total REE (1 M HCl), and the increase of REE recovery yield at 50 V FJH. Y_0 represents the REE recovery yield by directly leaching the e-waste raw materials. Y represents the REE recovery yield by leaching the activated e-waste. The error bars in A and B denote the standard deviation where N = 3.

The optimized FJH voltage for recovering REE from e-waste is 50 V. Too high voltage, which leads to too high temperature, might result in the evaporative loss of REE. The acid leachable content of total REE from the activated e-waste is ~95 ppm, corresponding to ~156% of the leachable REE content from e-waste raw materials.

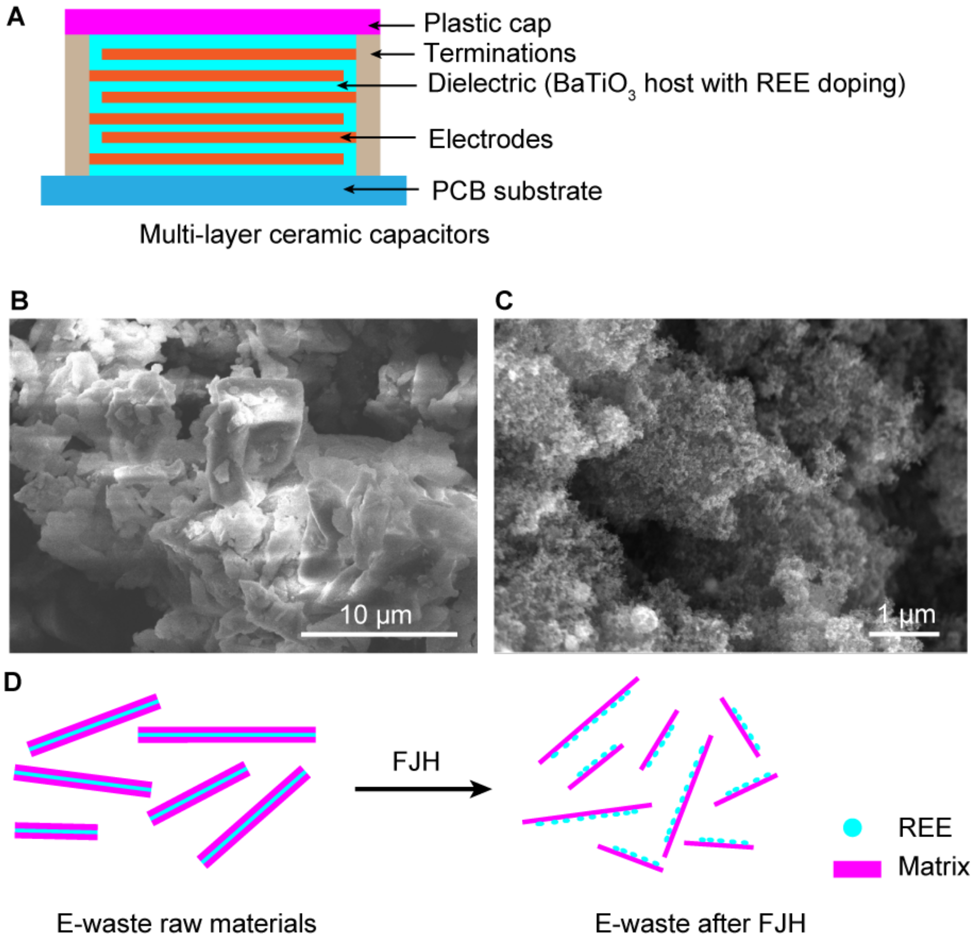

Fig. S14. Mechanism of the improved REE recovery yield in e-waste by FJH activation. (**A**) Structure scheme of the multi-layer ceramic capacitors. (**B**) SEM image of the e-waste after crushing. (**C**) SEM image of the e-waste after FJH. (**D**) Scheme of the morphology change of the e-waste during the FJH process.

The REE are widely used in modern electronics as strong magnets, ceramic capacitors, *etc*. Most electronic devices have the laminated structures, *e.g.*, the multilayer ceramic capacitors (*44*) (fig. S14A), and the REE are covered by the separator and protection layer of plastics and ceramics. The laminated structure makes it difficult for REE leaching by preventing the exposure of REE species to leachant. Even after grinding, the particle size of the e-waste is large (fig. S14B). After the FJH process, the laminated structures are broken (fig. S14C to D), which affords the acid leachable REE.

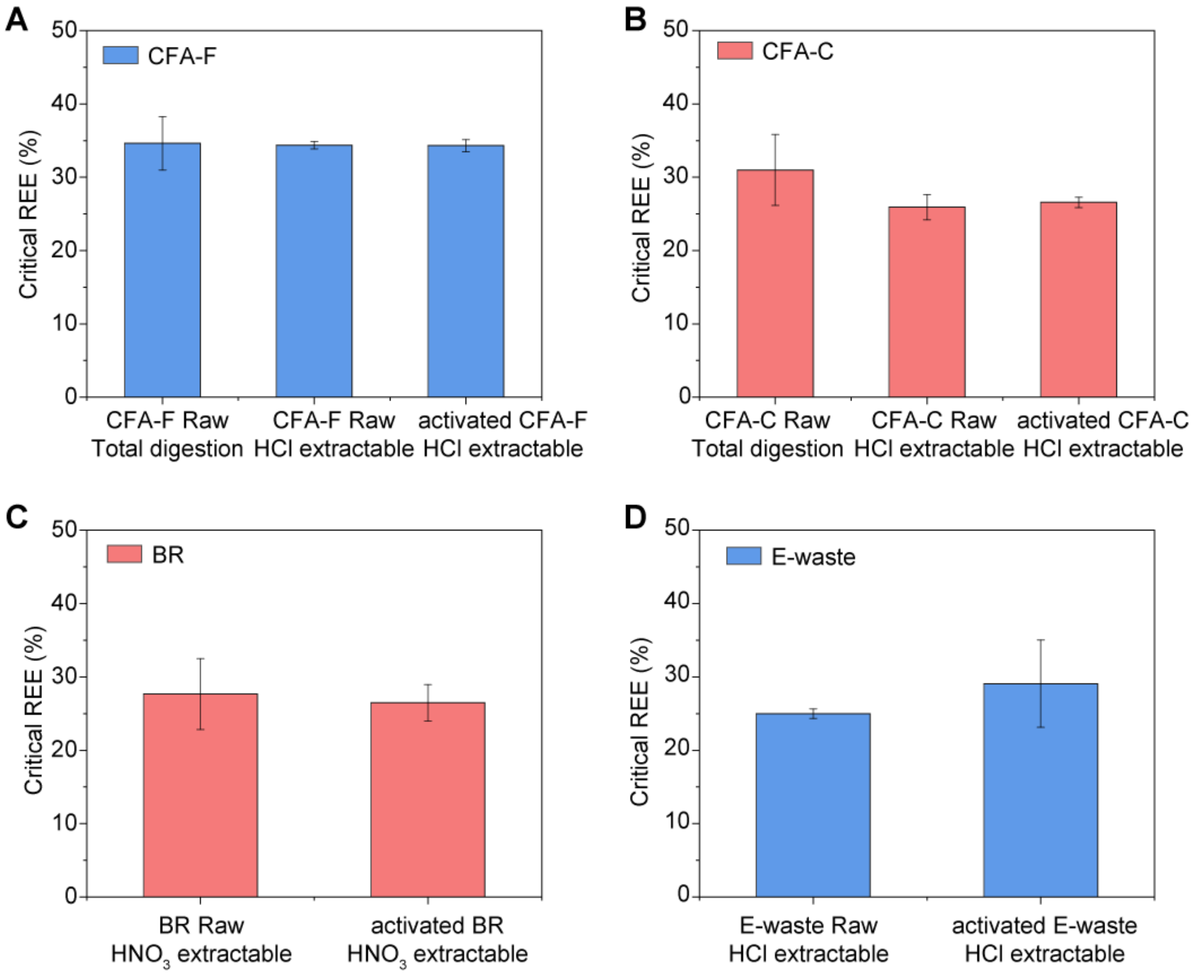

Fig. S15. The percentage of critical REE in total REE. (**A**) Percentage of critical REE in CFA-F, including the total digestion, HCl (1 M, 85 °C) extractable REE content in CFA-F Raw materials, and HCl extractable REE content in activated CFA-F. (**B**) Percentage of critical REE in CFA-C, including the total digestion, HCl (1 M, 85 °C) extractable REE content in CFA-C Raw materials, and HCl extractable REE content in activated CFA-C. (**C**) Percentage of critical REE in BR, including the HNO_3 (0.5 M, RT) extractable REE content in BR Raw materials, and HNO_3 extractable REE content in activated BR. (**D**) Percentage of critical REE in e-waste, including the HCl (1 M, 85 °C) extractable REE content in e-waste Raw materials, and HCl extractable REE content in activated e-waste. The critical REE include Y, Nd, Eu, Tb, and Dy. All error bars in (A) to (D) denote standard deviation where N = 3.

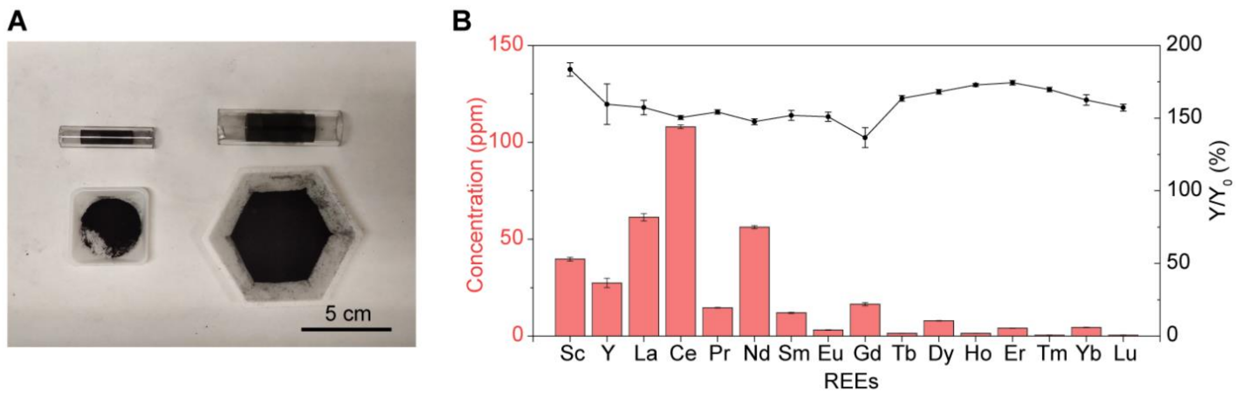

Fig. S16. Scaling up of the electrothermal activation process by using CFA-C as an example. (**A**) Picture of the small sample with mass of m_0 = 200 mg and quartz tube diameter of D_0 = 8 mm (left), and larger sample with mass of m_1 = 2 g and tube diameter of D_1 = 16 mm (right). (**B**) Acid leachable REE contents (1 M HCl, 85 °C) in activated CFA-C with the mass of m_1 = 2 g, and the increase of recovery yield. Y_0 represents the REE recovery yield by directly leaching the CFA-C raw materials. Y represents the REE recovery yield by leaching the activated CFA-C. The error bars in (B) denote the standard deviation where N = 3.

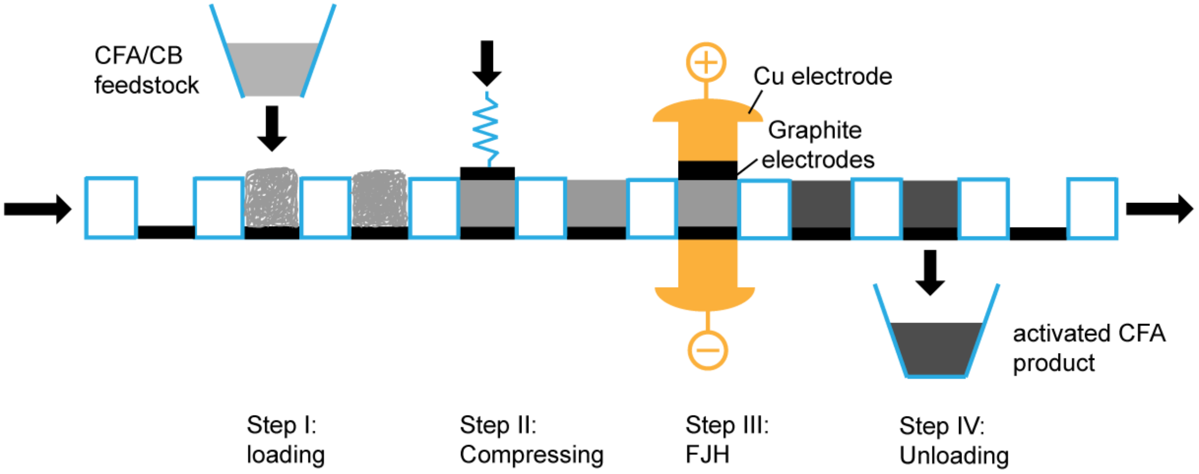

Fig. S17. Conceptual prototype design of the continuous FJH reactor.
The continuous production process consists of four steps. First, the mixture of CFA/CB feedstock is loaded onto the chamber on the conveyor belt. Secondly, the sample is compressed to a specific resistance. Thirdly, the sample undergoes the FJH reaction. Lastly, the activated CFA product is collected.

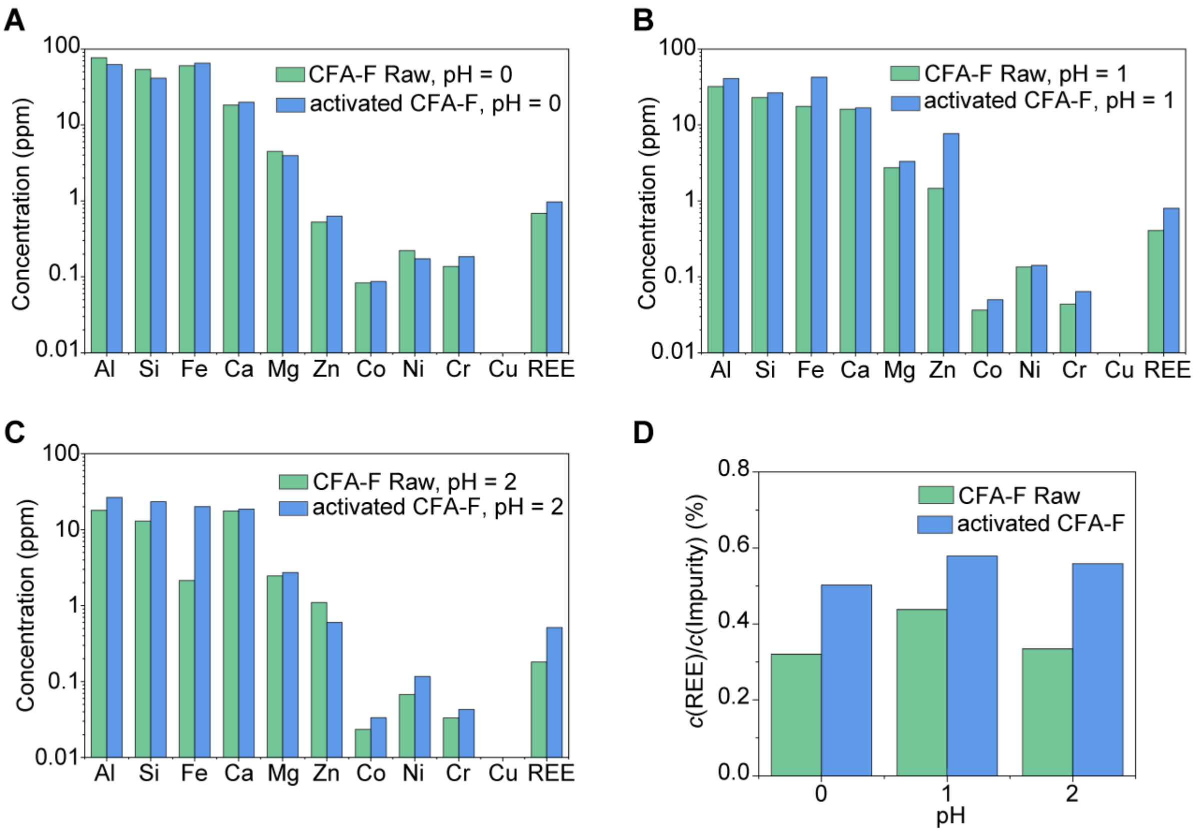

Fig. S18. Impurities in the REE-containing leachate from CFA-F. (**A**) Concentrations of metal impurities and REE in the leachate from CFA-F raw materials and activated CFA-F (1 M HCl, 85 °C). (**B**) Concentrations of metal impurities and REE in the leachate from CFA-F raw materials and activated CFA-F (0.1 M HCl, 85 °C). (**C**) Concentration of metal impurities and REE in the leachate from CFA-F raw materials and activated CFA-F (0.01 M HCl, 85 °C). (**D**) The ratio of REE content and metal impurities content in the leachate from CFA-F raw materials and activated CFA-F in leachant with different pH.

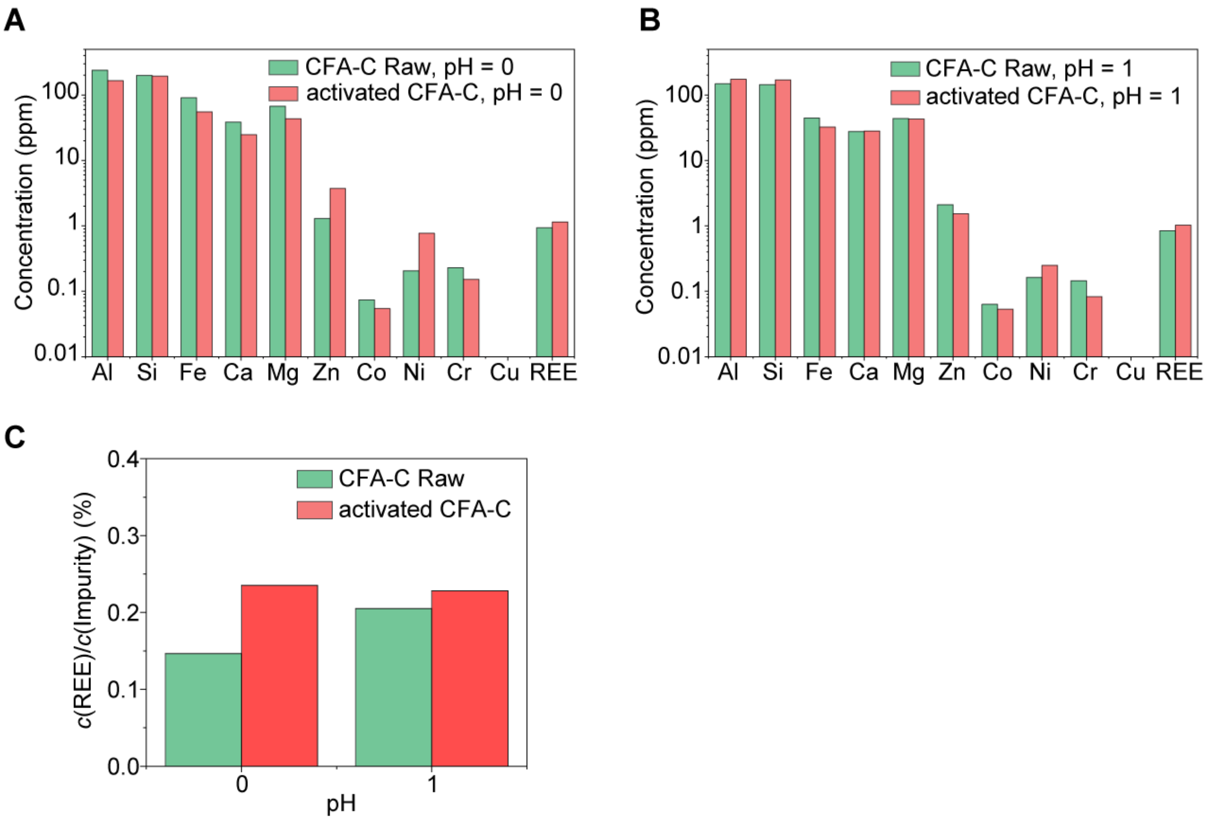

Fig. S19. Impurities in the REE-containing leachate from CFA-C. (**A**) Concentrations of metal impurities and REE in the leachate from CFA-C raw materials and activated CFA-C (1 M HCl, 85 °C). (**B**) Concentrations of metal impurities and REE in the leachate from CFA-F raw materials and activated CFA-C (0.1 M HCl, 85 °C). (**C**) The ratio of REE content and impurities content in the leachate from CFA-C raw materials and activated CFA-C in leachant with different pH.

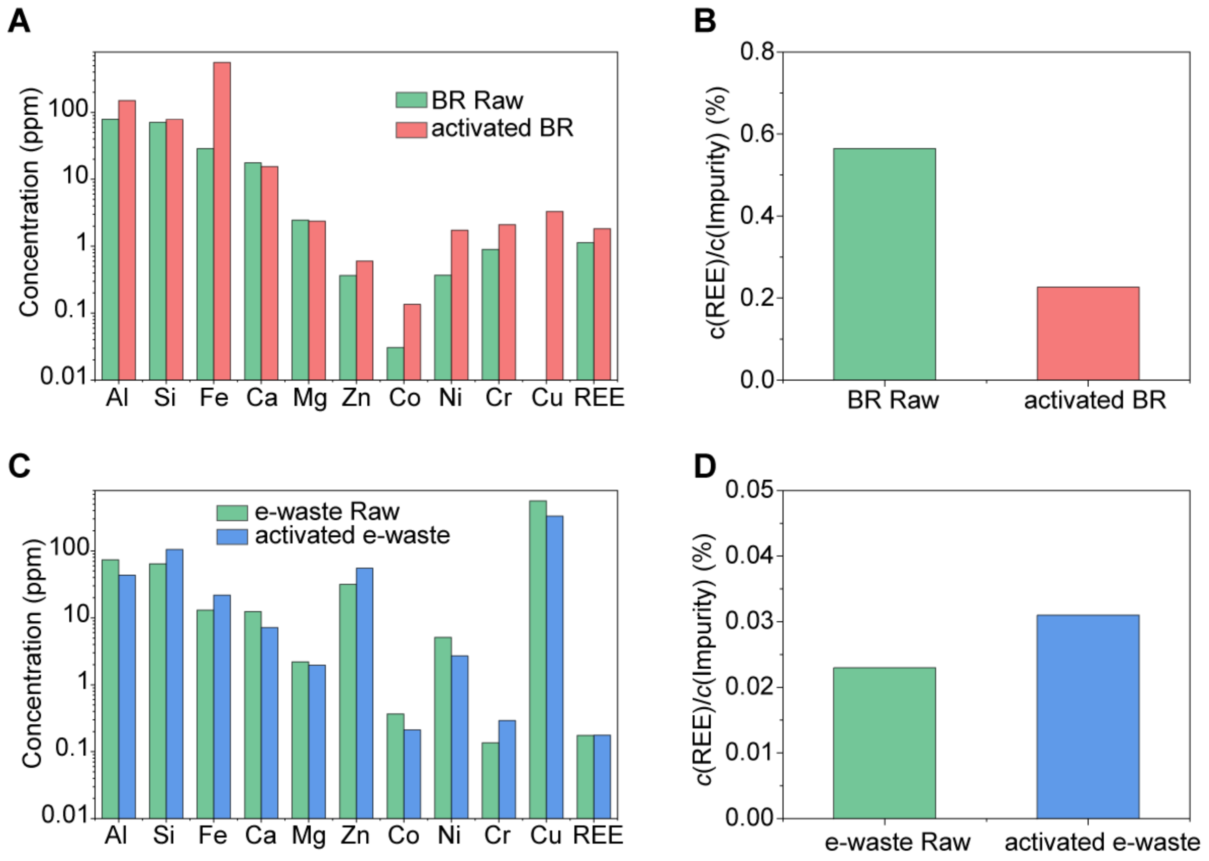

Fig. S20. Impurities in the REE-containing leachate from BR and e-waste. (**A**) Concentrations of metal impurities and REE in the leachate from BR raw materials and activated BR (0.5 M HNO_3, 85 °C). (**B**) The ratio of REE content and impurities content in the leachate from BR raw materials and activated BR. (**C**) Concentrations of metal impurities and REE in the leachate from e-waste raw materials and activated e-waste (1 M HCl, 85 °C). (**D**) The ratio of REE content and impurities content in the leachate from e-waste raw materials and activated e-waste.

Table S1. The FJH parameters for activation of secondary wastes.

Precursors	Mass Ratio	Mass (mg)	Resistance (Ω)	Voltage (V)	Time (s)	Mass after FJH (mg)
CFA-F:CB	2:1	200	1.0	50	1	190
CFA-F:CB	2:1	200	1.0	80	1	138
CFA-F:CB	2:1	200	1.0	100	1	115
CFA-F:CB	2:1	200	1.0	120	1	157
CFA-F:CB	2:1	200	1.0	150	1	88
CFA-C:CB	2:1	200	1.0	120	1	182
BR:CB	2:1	200	1.2	50	1	190
BR:CB	2:1	200	1.2	80	1	174
BR:CB	2:1	200	1.2	100	1	162
BR:CB	2:1	200	1.2	120	1	159
BR:CB	2:1	200	1.2	150	1	152
PCB:CB	2:1	200	1.0	50	1	162
PCB:CB	2:1	200	2.0	100	1	185
PCB:CB	2:1	200	2.0	120	1	98

Table S2. Thermal decomposition temperature of representative REE phosphates.

Material	Reaction	ΔH ($kJ\ mol^{-1}$)	ΔS ($J\ mol^{-1}\ K^{-1}$)	Temp (°C)
$LaPO_4$	$LaPO_4 = 1/2La_2O_3 + PO_2 + 1/4O_2$	817.65	260.45	2866
$CePO_4$	$CePO_4 = 1/2Ce_2O_3 + PO_2 + 1/4O_2$	785.8	256.3	2793
$PrPO_4$	$PrPO_4 = 1/2Pr_2O_3 + PO_2 + 1/4O_2$	798.8	269.8	2688
$NdPO_4$	$NdPO_4 = 1/2Nd_2O_3 + PO_2 + 1/4O_2$	780.85	262.1	2706
$SmPO_4$	$SmPO_4 = 1/2Sm_2O_3 + PO_2 + 1/4O_2$	774.3	256.5	2746
$EuPO_4$	$EuPO_4 = 1/2Eu_2O_3 + PO_2 + 1/4O_2$	765.0	266.7	2595
$GdPO_4$	$GdPO_4 = 1/2Gd_2O_3 + PO_2 + 1/4O_2$	766.4	253.9	2745

Note: The decomposition reaction, $REEPO_4 = 1/2REE_2O_3 + PO_2 + 1/4O_2$, is used for all REE phosphates (*30*). The standard molar enthalpies and standard molar entropies of REE phosphates are from the literature (*62*), and these constants of REE oxides, PO_2, and O_2 are from the CRC Handbook of Physics and Chemistry (*63*).

Table S3. Gibbs free energy change and solubility product constants (K_{sp}) of REE metals, oxides, and phosphates dissolution reactions at 25 °C.

Materials	Reaction	ΔG	$Log_{10}K_{sp}$
Sc	$Sc + 3H^+ = Sc^{3+} + 3/2H_2$	-614.2 kJ mol^{-1}	107.61
Sc_2O_3	$1/2Sc_2O_3 + 3H^+ = Sc^{3+} + 3/2H_2O$	-32.61 kJ mol^{-1}	5.71
$ScPO_4$	$ScPO_4 = Sc^{3+} + PO_4^{3-}$	---	-26.96
Y	$Y + 3H^+ = Y^{3+} + 3/2H_2$	-693.8 kJ mol^{-1}	121.56
Y_2O_3	$1/2Y_2O_3 + 3H^+ = Y^{3+} + 3/2H_2O$	-141.19 kJ mol^{-1}	24.74
YPO_4	$YPO_4 = Y^{3+} + PO_4^{3-}$	---	-25.02
La	$La + 3H^+ = La^{3+} + 3/2H_2$	-683.7 kJ mol^{-1}	119.79
La_2O_3	$1/2La_2O_3 + 3H^+ = La^{3+} + 3/2H_2O$	-186.49 kJ mol^{-1}	32.67
$LaPO_4$	$LaPO_4 = La^{3+} + PO_4^{3-}$	---	-25.7
Ce	$Ce + 3H^+ = Ce^{3+} + 3/2H_2$	-672 kJ mol^{-1}	117.74
Ce_2O_3	$1/2Ce_2O_3 + 3H^+ = Ce^{3+} + 3/2H_2O$	-174.59 kJ mol^{-1}	30.59
$CePO_4$	$CePO_4 = Ce^{3+} + PO_4^{3-}$	---	-26.2
Pr	$Pr + 3H^+ = Pr^{3+} + 3/2H_2$	-679.1 kJ mol^{-1}	118.98
Pr_2O_3	$1/2Pr_2O_3 + 3H^+ = Pr^{3+} + 3/2H_2O$	-174.89 kJ mol^{-1}	30.64
$PrPO_4$	$PrPO_4 = Pr^{3+} + PO_4^{3-}$	---	-26.4
Nd	$Nd + 3H^+ = Nd^{3+} + 3/2H_2$	-671.6 kJ mol^{-1}	117.67
Nd_2O_3	$1/2Nd_2O_3 + 3H^+ = Nd^{3+} + 3/2H_2O$	-166.89 kJ mol^{-1}	29.24
$NdPO_4$	$NdPO_4 = Nd^{3+} + PO_4^{3-}$	---	-26.2
Sm	$Sm + 3H^+ = Sm^{3+} + 3/2H_2$	-666.6 kJ mol^{-1}	116.79
Sm_2O_3	$1/2Sm_2O_3 + 3H^+ = Sm^{3+} + 3/2H_2O$	-154.99 kJ mol^{-1}	27.16
$SmPO_4$	$SmPO_4 = Sm^{3+} + PO_4^{3-}$	---	-26.1
Eu	$Eu + 3H^+ = Eu^{3+} + 3/2H_2$	-574.1 kJ mol^{-1}	100.58
Eu_2O_3	$1/2Eu_2O_3 + 3H^+ = Eu^{3+} + 3/2H_2O$	-151.39 kJ mol^{-1}	26.52
$EuPO_4$	$EuPO_4 = Eu^{3+} + PO_4^{3-}$	---	-25.9
Gd	$Gd + 3H^+ = Gd^{3+} + 3/2H_2$	-661 kJ mol^{-1}	115.81
Gd_2O_3	$1/2Gd_2O_3 + 3H^+ = Gd^{3+} + 3/2H_2O$	-134.79 kJ mol^{-1}	23.62
$GdPO_4$	$GdPO_4 = Gd^{3+} + PO_4^{3-}$	---	-25.6
Tb	$Tb + 3H^+ = Tb^{3+} + 3/2H_2$	-651.9 kJ mol^{-1}	114.21
Tb_2O_3	$1/2Tb_2O_3 + 3H^+ = Tb^{3+} + 3/2H_2O$	-119.19 kJ mol^{-1}	20.88
$TbPO_4$	$TbPO_4 = Tb^{3+} + PO_4^{3-}$	---	-25.3
Dy	$Dy + 3H^+ = Dy^{3+} + 3/2H_2$	-665 kJ mol^{-1}	116.51
Dy_2O_3	$1/2Dy_2O_3 + 3H^+ = Dy^{3+} + 3/2H_2O$	-134.94 kJ mol^{-1}	23.64
$DyPO_4$	$DyPO_4 = Dy^{3+} + PO_4^{3-}$	---	-25.1
Ho	$Ho + 3H^+ = Ho^{3+} + 3/2H_2$	-673.7 kJ mol^{-1}	118.03
Ho_2O_3	$1/2Ho_2O_3 + 3H^+ = Ho^{3+} + 3/2H_2O$	-133.84 kJ mol^{-1}	23.45
$HoPO_4$	$HoPO_4 = Ho^{3+} + PO_4^{3-}$	---	-25.0
Er	$Er + 3H^+ = Er^{3+} + 3/2H_2$	-669.1 kJ mol^{-1}	117.23
Er_2O_3	$1/2Er_2O_3 + 3H^+ = Er^{3+} + 3/2H_2O$	-120.44 kJ mol^{-1}	21.10
$ErPO_4$	$ErPO_4 = Er^{3+} + PO_4^{3-}$	---	-25.1
Tm	$Tm + 3H^+ = Tm^{3+} + 3/2H_2$	-662 kJ mol^{-1}	115.98
Tm_2O_3	$1/2Tm_2O_3 + 3H^+ = Tm^{3+} + 3/2H_2O$	-120.44 kJ mol^{-1}	21.10
$TmPO_4$	$TmPO_4 = Tm^{3+} + PO_4^{3-}$	---	-25.0

Continuing table

**Materials**	**Reaction**	**ΔG**	**$Log_{10}K_{sp}$**
Yb	$Yb + 3H^+ = Yb^{3+} + 3/2H_2$	-644 kJ mol^{-1}	112.83
Yb_2O_3	$1/2Yb_2O_3 + 3H^+ = Yb^{3+} + 3/2H_2O$	-136.34 kJ mol^{-1}	23.89
$YbPO_4$	$YbPO_4 = Yb^{3+} + PO_4^{3-}$	---	-24.8
Lu	$Lu + 3H^+ = Lu^{3+} + 3/2H_2$	-628 kJ mol^{-1}	110.03
Lu_2O_3	$1/2Lu_2O_3 + 3H^+ = Lu^{3+} + 3/2H_2O$	-89.19 kJ mol^{-1}	15.63
$LuPO_4$	$LuPO_4 = Lu^{3+} + PO_4^{3-}$	---	-24.7

Note: For REE metals, the $log_{10}K_{sp}$ values are calculated from the free energy of formation constants in the CRC Handbook of Physics and Chemistry (*63*). For REE oxides, the $log_{10}K_{sp}$ values are calculated from the free energy of formation constants reported in the NBS tables of thermodynamic properties (*64*). There is no free energy of formation for Pr_2O_3, Gd_2O_3, and Tb_2O_3 in the NBS table; for these oxides, the $log_{10}K_{sp}$ values are calculated based on the enthalpy of formation and standard entropy for element from NBS table (*64*), and standard entropy of the oxides from a literature (*65*). For REE phosphates, the $log_{10}K_{sp}$ values are reported in literatures for $ScPO_4$ (*66*) and other REE phosphates (*67*). Generally, the REE phosphates are hard to dissolve and have the $log_{10}K_{sp}$ values in the range of -24 to -27. These values are significantly smaller than those of REE metals and REE oxides. As a result, REE metals and REE oxides are much more extractable by acid than the REE phosphate counterparts.

Table S4. The XPS peak fitting of La and Y.

**Samples**	**Element**	**Position (eV), peak**	**Chemical state, Chemical bond**
La_2O_3	La	834.8, La $3d_{5/2}$	+3, La-O
		838.2, satellite	+3, La-O
La_2O_3 after FJH	La	834.8, La $3d_{5/2}$	+3, La-O
		836.0, La $3d_{5/2}$	0, La-La
		838.1, satellite	+3, La-O
		839.6, satellite	0, La-La
Y_2O_3	Y	157.4, Y $3d_{5/2}$	+3, Y-O
		159.4, Y $3d3_{/2}$	+3, Y-O
Y_2O_3 after FJH	Y	156.4, Y $3d_{5/2}$	0, Y-Y
		157.5, Y $3d_{5/2}$	+3, Y-O
		158.5, Y $3d_{3/2}$	0, Y-Y
		159.6, Y $3d_{3/2}$	+3, Y-O
